# Genome-Wide Investigation of the Cysteine Synthase Gene Family Shows That Overexpression of *CSase* Confers Alkali Tolerance to Alfalfa (*Medicago sativa* L.)

**DOI:** 10.3389/fpls.2021.792862

**Published:** 2022-01-04

**Authors:** Yuying Yuan, Tingting Song, Jinqiu Yu, Wenkai Zhang, Xiangyin Hou, Zelai Kong Ling, Guowen Cui

**Affiliations:** Department of Grassland Science, College of Animal Science and Technology, Northeast Agricultural University, Harbin, China

**Keywords:** alfalfa, *CSase* gene family, genome-wide analysis, gene overexpression, alkali stress

## Abstract

Alfalfa is widely grown worldwide as a perennial high-quality legume forage and as a good ecological landcover. The cysteine synthase (CSase) gene family is actively involved in plant growth and development and abiotic stress resistance but has not been systematically investigated in alfalfa. We identified 39 *MsCSase* genes on 4 chromosomes of the alfalfa genome. Phylogenetic analysis demonstrated that these genes were clustered into six subfamilies, and members of the same subfamily had similar physicochemical properties and sequence structures. Overexpression of the *CSase* gene in alfalfa increased alkali tolerance. Compared with control plants, the overexpression lines presented higher proline, soluble sugars, and cysteine and reduced glutathione contents and superoxide dismutase and peroxidase activities as well as lower hydrogen peroxide and superoxide anion contents after alkali stress. The relative expression of γ-glutamyl cysteine synthetase gene (a downstream gene of *CSase*) in the overexpression lines was much higher than that in the control line. The *CSase* gene enhanced alkalinity tolerance by regulating osmoregulatory substances and improving antioxidant capacity. These results provide a reference for studying the CSase gene family in alfalfa and expanding the alkali tolerance gene resources of forage plants.

## Introduction

Cysteine is the first organic substance in plants found to contain both sulfur and nitrogen and is a precursor of sulfur-containing metabolites such as methionine (Takahashi et al., 2011), glutathione (GSH) and Fe-S clusters, which play an important role in plant development and metabolic processes (Droux, 2004; Van Hoewyk et al., 2008). The synthesis of cysteine can be roughly divided into the absorption and reduction of elemental sulfur (Kopriva, 2006; Davidian and Kopriva, 2010). Cysteine synthase (CSase) is involved in the final step of cysteine synthesis; this enzyme catalyzes the synthesis of cysteine from H_2_S and O-acetylserine (OAS) (Jez and Dey, 2013; Romero et al., 2014).

The *CSase* gene is often referred to as the O-acetylserine(thiol)lyase gene (*OAS-TL*) and belongs to the CSase gene family (Droux et al., 1998; Wirtz and Hell, 2006; Alvarez et al., 2010a). The CSase gene family was previously identified in Arabidopsis (*Arabidopsis thaliana*) and was found to comprise nine genes divided into five subfamilies, all of whose members contain PLP-binding sites (PXXSVKDR) that are highly conserved across species (Yamaguchi et al., 2000). However, cytosolic *OAS-A1*, plastidial *OAS-B*, and mitochondrial *OAS-C* were identified as the three *OASTLs* that were also expressed at relatively high levels and interacted with ser acetyltransferase (*SAT*) (Bonner et al., 2005; Heeg et al., 2008; Jez and Dey, 2013). In addition, *CYS-D1* and *CYS-D2* also have weak cysteine synthesis functions in mitochondria (Yamaguchi et al., 2000). CSases compose a protein family whose members have multiple functions, and *CSase* genes in different tissue sites may have different functions. For example, L-cysteine desulfhydrase 1 (*DES1*) in the cytoplasm has L-cysteine desulfhydrase activity, sulfocysteine synthase (*SCS)* in the chloroplast encodes S-thiocysteine synthase, and the mitochondrial enzyme *CAS-C1* has β-cyanoalanine synthase (CAS) activity (Alvarez et al., 2010a; Bermúdez et al., 2010). Recently, it has also been shown that *CSase* genes are involved in environmental stress responses such as responses to high-salt conditions and heavy metals, and overexpression of *CSase* genes has been shown to increase the ability of plants to adapt to oxidative stress (Ning et al., 2009; Xie et al., 2012). Moreover, a wide range of defense compounds that can respond to adverse environments use cysteine as a precursor (Alvarez et al., 2010b). Taking the GSH metabolic pathway as an example, the synthesis of GSH as an antioxidant molecule is restricted by cysteine, and in turn GSH is a precursor for the synthesis of phytochelatins (PCs), thiolated peptides involved in the detoxification of heavy metals (Cui et al., 2012, 2014). When plants need to enhance GSH biosynthesis under heavy metal stress, *CSase* can increase cysteine production and subsequently affect the synthesis of downstream substances and achieve improved plant tolerance.

The earliest report of the *CYS-C1* gene in Arabidopsis involved a CAS that converts cyanide and cysteine to β-cyanoalanine and H_2_S in mitochondria. *CYS-C1* and *Cys-C* act together to complete the cyclic pathway of cyanide detoxification (García et al., 2010; Álvarez et al., 2012b). However, the activity of *CYS-C1* during cysteine synthesis is also relatively high (Hatzfeld et al., 2000), and *CYS-C1* is considered a member of the CSase gene family (Watanabe et al., 2008). Since *SAT* and *CSase* interact for efficient synthesis of cysteine, authentic *CSase* can interact with *SAT* (Droux et al., 1998; Romero et al., 2014). Moreover, the *SlOAS7* gene in the CYS-C subfamily was found to interact with *SAT* in tomato (Liu et al., 2018). Based on the above information, it is speculated that CYS-C subfamily members may also be true CSases.

Alfalfa (*Medicago sativa* L.), which is widely grown in Asia, Europe, and America, is a high-quality perennial forage plant of the legume family; alfalfa is high yielding and rich in nutrients and is one of the most important forage species for healthy and efficient livestock breeding. The CSase gene family has been extensively studied in many species, and genome-wide analyses have identified members of the CSase gene family in Arabidopsis (Yamaguchi et al., 2000), tomato (*Solanum lycopersicum* L.) (Liu et al., 2018), foxtail millet (*Setaria italica* (L.) P. Beauvois) (Liu et al., 2019), and sorghum (*Sorghum bicolor*) (Akbudak et al., 2018), but our knowledge of the CSase gene family in forage crop species such as alfalfa is still limited. The recently published genome of alfalfa (cultivar Xinjiangdaye) provides an important resource for further molecular studies of this species (Chen et al., 2020). Previous work by our group found that this gene responds to alkali stress (Song et al., 2017, 2021). Based on this information, a total of 39 *CSase* genes were identified and classified into 6 subfamilies in this study, and bioinformatic analyses including phylogenetic analysis, motif composition analysis, and gene duplication analysis were performed to provide a theoretical basis for clarifying the evolutionary history and biological functions of the members of this gene family. In addition, we successfully cloned a *CSase* gene (belonging to the CYS-C1 subfamily) from alfalfa, transferred it into alfalfa, and analyzed its potential function. The results showed that this gene encodes a protein that promotes cysteine synthesis and improves the alkalinity tolerance of overexpression lines by increasing the antioxidant capacity of the plant.

## Materials and Methods

### Identification of Cysteine Synthase Gene Family Members in Alfalfa

The sequences of the nine identified *AtCSase* genes were obtained from the NCBI database.^1^ The *M. sativa* Xinjiangdaye genome sequence was downloaded from a website.^2^ *MsCSases* were identified by two rounds of BLASTP. A hidden Markov model (HMM) was used by Pfam 31.1^3^ to ensure that the PF00291 domain was retained, and DNAMAN was used for sequence comparison searches for the PLP-binding site (PXXSVKDR) in alfalfa.

### Phylogenetic Analysis and Multiple Sequence Alignment

A phylogenetic tree was generated by MEGA 5 using the NJ method, with 1,000 bootstrap replicates. Multiple sequence alignments of CSases were created with ClustalX.

### Analysis of Conserved Motifs and Conserved Domains

The conserved motif structures within the CSase sequences were identified by MEME Suite Version 5.2.0^4^ with the following parameters: zero or one occurrence per sequence of site distribution, a maximum of 10 misfits and a maximum width of motif between 6 and 50. NCBI Batch CD-Search^5^ was used to analyze the conserved domains of the CSase proteins, after which the domains were visualized by TBtools.

### Vector Construction and Plant Transformation

We used the *CSase* gene of *M. truncatula* (*Medtr7g078070.1*) as a reference sequence to clone the *CSase* gene of *M. sativa*. Transient expression vectors for tobacco and overexpression vectors for alfalfa were constructed by the one-step cloning method. Then, the expression vectors were transformed into *Agrobacterium rhizogenes* by using the freeze-thaw method. The *CSase* gene was transformed into alfalfa *via* Agrobacterium mediation using the cotyledon method, and regenerated alfalfa plants were obtained. The *bar* gene detection method and fluorescence quantitative analysis technology were used to screen overexpression plants. The sequences of the primers used are shown in Supplementary Table 1.

### Plant Growth Conditions and Treatments

*Nicotiana benthamiana* plants were grown in plastic pots filled with vermiculite. Approximately 1-month-old seedlings were used for transient expression. *M. sativa* Longmu 801 was used in this study. Softwood cuttings from the CK line and overexpression lines OV#L11, OV#L12, and OV#L13 were transplanted into plastic pots containing vermiculite (one plant per pot). All the plants were grown under a 16 h light/8 h dark photoperiod under a day/night temperature cycle of 22°C/18°C. Hoagland solution (1/10 strength) was applied to the plants every 3 days.

For NaHCO_3_ treatment, 150 mM NaHCO_3_ was applied for 5 d, and a 5 d recovery period was selected as the best condition for identifying stress phenotypes. Samples were taken at 0, 1, 6, 12, 24, 48 h, and 5 d after the beginning of the treatment. Three biological replicates were included per line.

### Subcellular Localization Analysis

To explore the subcellular localization of CSase proteins, we constructed a transient expression vector. *CSase* gene was inserted downstream from the double CaMV 35S promoter in the pCAMBIA1300 vector. The pCAMBIA1300 vector carries GFP gene. The sequences of the primers used are listed in Supplementary Table 1. The resulting vector was introduced into the *Agrobacterium tumefaciens* strain GV3101. We used a syringe to infiltrate *Agrobacterium tumefaciens* strain GV3101 containing a tobacco transient expression vector into 1-month-old tobacco leaves. After infiltration, the plants were cultivated for 72 h under dark conditions. The fluorescence signal in the infested tobacco leaves was subsequently observed by confocal microscopy.

### Determination of Physiological Indicators and Expression Analysis of Related Genes

Physiological traits including GSH, cysteine, Pro, and MDA contents and SOD and POD activities were measured using reagent kits (Nanjing Jiancheng Bioengineering Institute, Nanjing, China). The instructions of the kits were followed for specific test procedures.

For qRT-PCR, total RNA was isolated from alfalfa samples using an RNeasy Plant Mini Kit (CWBIO, Jiangsu, China), and cDNA was synthesized using a kit (Vazyme). qRT-PCR was used to analyze the relative expression levels of alfalfa CSase-responsive genes. The *GADPH* gene was used as a reference. The sequences of the primers used are shown in Supplementary Table 1.

### Statistical Analyses

To determine significance, all statistical analyses were performed by using Microsoft Excel.

## Results

### Identification of MsCSase Genes in Alfalfa

**FIGURE 1:**
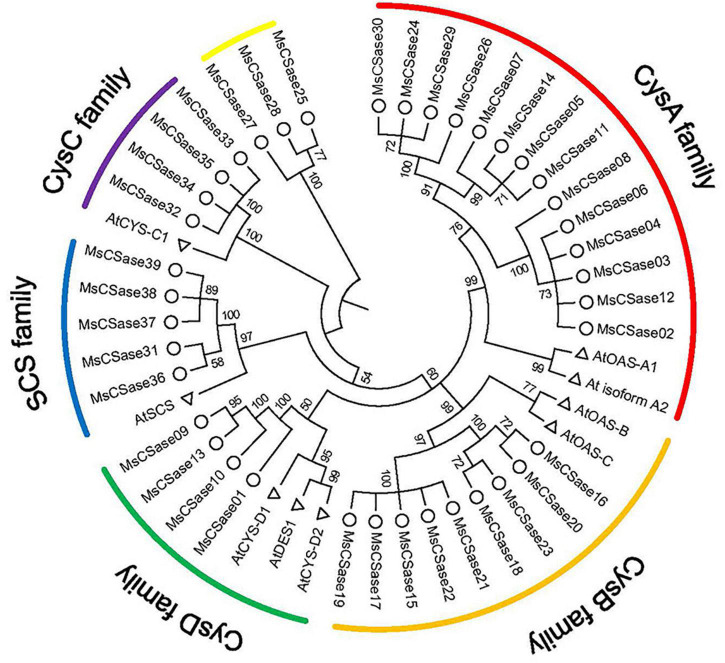
Phylogenetic tree generated using the NJ method. The tree represents relationships among CSases of alfalfa and Arabidopsis. The different colored arcs indicate different CSase subfamilies. Δ and ° represent *CSase* genes in alfalfa and Arabidopsis, respectively. The number represents the bootstrap value of the node. Larger node values imply a higher confidence level.

First, a total of 39 *MsCSase* gene sequences were retrieved from alfalfa using BLAST, PF00291 domain and PLP-binding site (PXXSVKDR) searches and named *MsCSase01* to *MsCSase39* according to their chromosome locations (Supplementary Table 2). They were unevenly mapped onto chromosomes 1, 4, 5, and 7, which contained 14, 9, 7, and 9 genes, respectively (Supplementary Table 2). Information about their coding DNA sequences (CDS) and resulting protein sequences are presented in Supplementary Table 3. Then, characterization of the proteins revealed that the predicted isoelectric points (pIs) of the MsCSase proteins ranged from 5.17 to 9.08 (Supplementary Table 2). Except for *MsCSase01*, *MsCSase31* and *MsCSase36*, the length and molecular mass did not widely vary (Supplementary Table 2). The phylogenetic tree results demonstrated that the MsCSase proteins could be divided into 6 subfamilies according to the clades and classification from Arabidopsis, including 14, 9, 5, 4, and 4 members in the CysA subfamily, CysB subfamily, SCS subfamily, CysD subfamily and CysC subfamily, respectively (Figure 1). Similar to that which occurred in a study in tomato (Liu et al., 2018), *MsCSase25*, *MsCSase27*, and *MsCSase28* were separated into a separate family and did not belong to the other five subfamilies. These results indicated that the characteristics and patterns of evolution in various species are more likely to differ.

### Structural Features and Synteny Analysis

**FIGURE 2:**
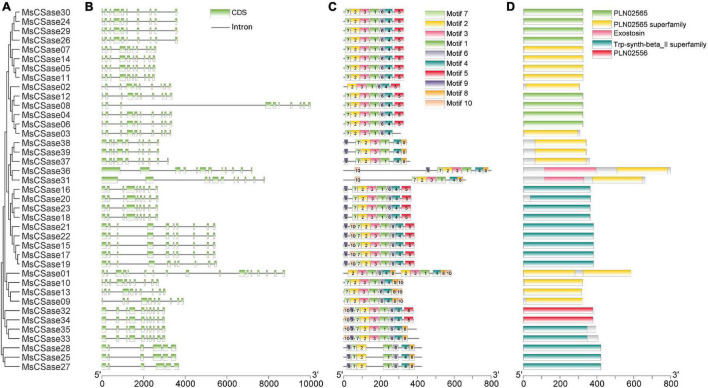
Architecture of conserved protein motifs, domains and gene structures among the MsCSase gene family members in alfalfa. **(A)** Phylogenetic tree of alfalfa MsCSase proteins constructed using MEGA 5 software. **(B)** Gene structure of the MsCSase gene family members. The green boxes indicate exons; the black lines indicate introns. **(C)** Motif composition of MsCSase proteins. The motifs, numbered 1–10, are displayed in different colored boxes. The results of a sequence logo analysis of the conserved motifs are provided in Supplementary Figure 1. The length of the protein can be estimated using the scale at the bottom. **(D)** The characterized domains of the *MsCSase* genes were identified. The different colors represent different domains.

Genetic structural diversity supported the phylogenetic groupings to some extent (Wei et al., 2016). Therefore, we analyzed the relationship between gene structure and phylogenetic clustering to gain insight into the evolution of the MsCSase gene family in alfalfa (Figures 2A,B). Gene structure analysis showed that genes within the same subfamily presented similar structures; for instance, the CysA subfamily members contained 10 exons, and the exon distribution of genes on the same branch was largely similar. In addition, the protein motif analysis (the conserved motifs *via* sequence logo are shown in Supplementary Figure 1) by MEME found a similar pattern and the order and distribution of the motifs were roughly similar among the members of the subfamilies (Figure 2C). These results supported the close evolutionary relationship of the classification of these MsCSase subfamilies. Moreover, the MsCSase genes had five characterized domains, including PLN02565, the PLN02565 superfamily, Trp-synth-beta_II superfamily and PLN02556 domains, all of which are related to cysteine synthesis (Figure 2D). Overall, the domain similarity suggested that these genes may have similar functions, but the differences in the activity of their encoded enzymes may be related to differences in gene structure and motifs between subfamily members.

To elucidate the mechanism through which the MsCSase gene family members in alfalfa expanded, gene duplication events were identified. Two pairs of tandem duplication genes (*MsCSase9/10*, *MsCSase22/23*) and 35 groups of synteny gene pairs in which *MsCSase07/24* were segmentally duplicated genes were identified by TBtools and MCScanX software (Supplementary Table 4). Duplicated genes were located on chromosomes 1, 5, and 10 (Supplementary Figure 2). Taken together, the results indicated that there was no obvious relationship between chromosome length and the number of genes. Some *MsCSase* genes may have been generated by gene duplications and tandem and segmental duplications contributed to the evolution of *MsCSases* in alfalfa.

### Molecular Cloning and Subcellular Localization of CSase

Using the *Medtr7g078070.1* gene of *Medicago truncatula* as a probe, we cloned the gene with accession number MK334208 named *CSase* from alfalfa (cultivar Longmu 801). Sequence analysis showed that the amino acid sequences of *MsCSas32*, *MsCSase34*, and *CSase* were nearly identical (Supplementary Figure 3); they were 99.91% similar at the nucleotide level, and only one nucleotide differed between *MsCSase34* and *CSase* (Supplementary Figure 4). Combining the results of the phylogenetic evolutionary tree analysis with these results, we determined that the *CSase* gene belonged to the CYS-C1 subfamily and was highly conserved in two different alfalfa varieties.

**FIGURE 3:**
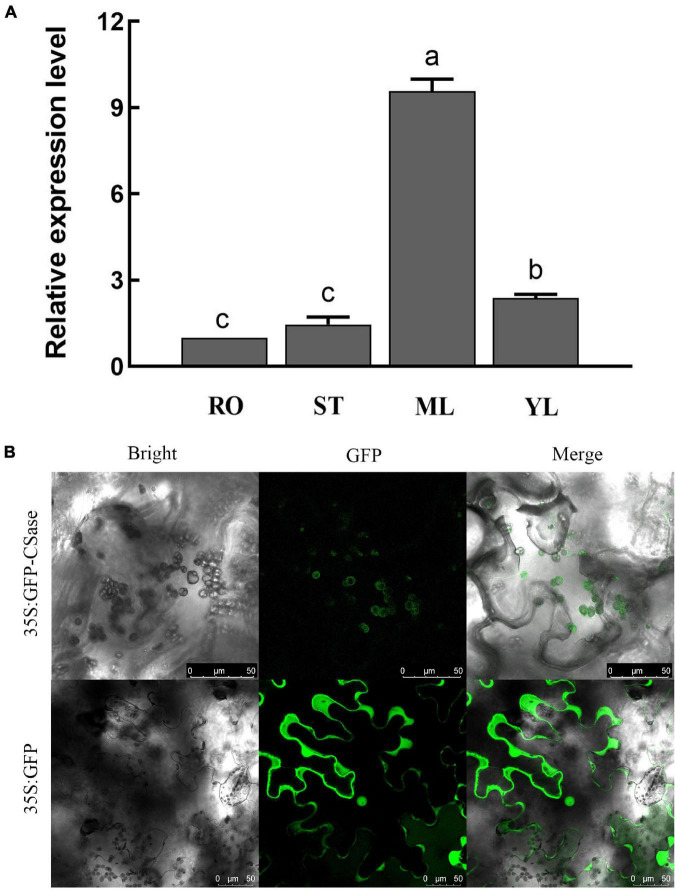
Expression pattern and subcellular localization of CSase. **(A)** Differential expression of representative *CSases* in different tissues according to qRT-PCR. RO, root; ST, stem; ML, mature leaf; and YL, young leaf. **(B)** Confocal laser scanning microscopy images of tobacco leaf cells expressing the CSase protein (35S:GFP-CSase) and GFP protein (35S:GFP). Scale bars = 50 μm.

qRT-PCR analysis of different alfalfa tissue parts revealed that the relative expression of *CSase* was higher in the leaves than in the other tissues (Figure 3A). Moreover, the relative expression in mature leaves was much higher than that in other tissues. To further clarify the location of gene activity, we evaluated the subcellular localization of *CSase*. We fused its ORF sequence without the terminal codon to GFP at the N-terminus under the control of the CaMV 35S promoter and ultimately transiently expressed it in tobacco (*N. benthamiana*) leaf epidermal cells. The results showed that the presence of the CSase–GFP fusion protein in the chloroplasts of the cells (Figure 3B). This finding also directly validates the accuracy of the qRT-PCR results.

### Overexpression of Cysteine Synthase Enhances Alkali Tolerance in Transgenic Alfalfa

**FIGURE 4:**
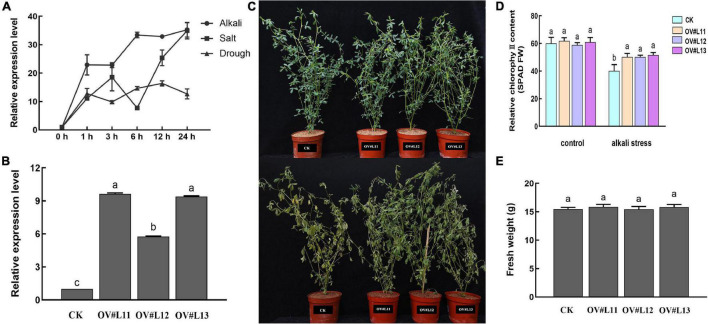
Overexpression of *CSase* confers alkalinity tolerance to alfalfa. **(A)** Relative expression of *CSase* under salt, alkali and drought stress. **(B)** Relative expression of *Bar* in different lines. **(C)** Phenotypic plots of different lines under normal growth and alkali conditions. **(D)** Tissue SPAD value of different lines under normal and alkali stress conditions. **(E)** Fresh weight of each line under normal conditions. The average of three independent samples was calculated and is shown, together with the standard error of the mean (bars) between samples. Different lowercase letters under the same conditions indicate that the difference is significant (*P* < 0.05).

qRT-PCR analysis under different abiotic stresses revealed that *CSase* could respond positively to salt, alkali and drought stresses in alfalfa, and the response to alkali stress was more pronounced than that to the other stressors (Figure 4A). To determine whether alfalfa alkalinity tolerance was altered by up-regulation of *CSase*, we constructed alfalfa overexpression vectors (Supplementary Figure 5) and transformed the CDS of *CSase* into the alfalfa cultivar Longmu 801 to obtain *CSase*-overexpressing transgenic lines. The glucosamine gene is a marker gene that is present only in the vector itself and not in alfalfa. *Bar* gene detection revealed successful infestation of alfalfa in response to a bacterial solution (Supplementary Figure 6). qRT-PCR analysis showed that the relative expression of the *MsCSase* gene in the control (CK) lines were much lower than that in the OV# L11-, OV# L12-, and OV#L13-overexpressed lines (Figure 4B). This indicates that the *CSase* gene was successfully overexpressed in alfalfa. We ultimately selected the CK line and overexpression lines OV#L11, OV#L12, and OV#L13 for subsequent experiments.

Under normal growth conditions, there was no significant difference in phenotype between the CK and overexpression lines, and the differences between the aboveground biomass fresh weight and the relative chlorophyll content were not significant. However, after 5 d of alkali stress, the leaf wilting of the overexpression lines was much lower than that of the CK (Figure 4C). The alkali treatment led to degradation of the relative chlorophyll content (Figure 4D), but the chlorophyll contents were still higher in the overexpression lines than in the CK line. Moreover, the difference in aboveground biomass between the lines was not significant (Figure 4E). Overall, overexpressing *CSase* provided increased tolerance to alkali stress in alfalfa.

**FIGURE 5:**
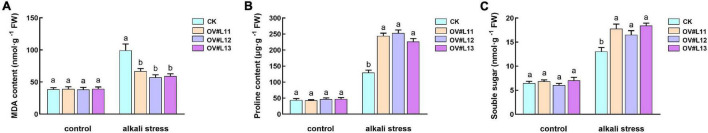
Contents of MDA **(A)**, Pro **(B)**, and SSs **(C)** in each line under normal conditions and after alkali stress. One-month-old alfalfa seedlings were subjected to 150 mM NaHCO_3_ solution for 5 days. The average of three independent samples was calculated and is shown, together with the standard error of the mean (bars) between samples. Different lowercase letters under the same conditions indicate that the difference is significant (*P* < 0.05).

To clarify the reason for the increased alkali tolerance of the *CSase* overexpression lines, we also measured the contents of malondialdehyde (MDA), proline (Pro) and soluble sugars (SSs) in the *CSase* overexpression lines and CK plants grown under normal and alkaline conditions. However, after alkali stress, the contents of MDA, Pro and SSs increased within each line, and the increase in MDA content in the overexpression line was significantly lower than that in CK, while the contents of osmoregulatory substances containing Pro and SSs were significantly higher than those in CK (Figures 5A–C). It can be hypothesized that, compared with the CK plants, the overexpression lines are alkaline tolerant due to their lower degree of membrane damage and higher accumulation of osmoregulatory substances.

### Cysteine Synthase Overexpression Increased the Cysteine and Glutathione Contents to Improve Alkali Tolerance

**FIGURE 6:**
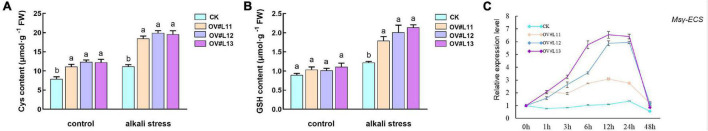
Contents of cysteine **(A)** and GSH **(B)** and the relative expression of the *Ms*γ*-ECS* gene **(C)** in each line under normal conditions and after alkali stress. One-month-old alfalfa seedlings were subjected to 150 mM NaHCO_3_ solution for 5 days. The average of three independent samples was calculated and is shown, together with the standard error of the mean (bars) between samples. Different lowercase letters under the same conditions indicate that the difference is significant (*P* < 0.05).

*CSases* have been shown to encode CSase proteins (Hatzfeld et al., 2000). The increase in CSase content promotes an increase in cysteine content in plants. In the present study, the cysteine content in the overexpression lines was significantly higher than that in CK under normal growth conditions, and the cysteine content under alkali stress was higher than that in the CK and tended to increase (Figure 6A). Accordingly, we speculated that cysteine plays a role in improving alkali tolerance in plants.

Based on our preliminary research, we found that cysteine is a precursor for the synthesis of antioxidant substances such as GSH (Droux, 2004; Wirtz and Droux, 2005; Kopriva, 2006) and that GSH plays an important role in enhancing antioxidant capacity and transducing redox-sensitive signals in plants (Cnubben et al., 2001; Matés et al., 2002; Pastori and Foyer, 2002). Therefore, we determined the GSH content and the relative expression of γ-glutamyl cysteine synthetase (γ*-ECS*) in each line. The results showed that the GSH content in the plants increased in response to alkali stress and that the overexpression lines contained more GSH (Figure 6B). The trends of the relative expression of γ*-ECS* in the overexpression and CK lines were similar, but the relative expression of γ*-ECS* was higher in the overexpression line than in the CK line (Figure 6C). Overexpression of *CSase* and *SAT* in tobacco also significantly increased the relative expression of γ*-ECSs* as well as the GSH content in the plants (Nakamura et al., 2014). Thus, the overexpression of the *CSase* gene could regulate downstream metabolic pathways, which led to an increase in the relative expression of the downstream γ*-ECS* gene, and the increase in cysteine content provided the possibility of an increase in GSH content. This also laid the foundation for the improvement in alkali tolerance in the overexpression line.

### Cysteine Synthase Overexpression Increased the Antioxidant Capacity of Transgenic Alfalfa

**FIGURE 7:**
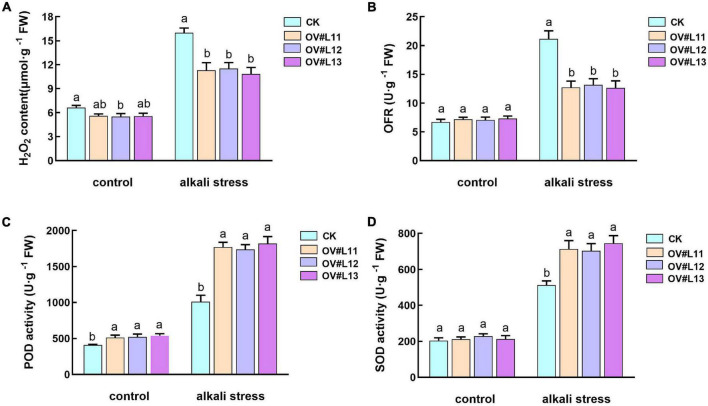
Content of H_2_O_2_ **(A)** and OFR **(B)** and the activities of POD **(C)** and SOD **(D)** in each line under normal conditions and after alkali stress. One-month-old alfalfa seedlings were subjected to 150 mM NaHCO_3_ solution for 5 days. The average of three independent samples was calculated and is shown, together with the standard error of the mean (bars) between samples. Different lowercase letters under the same conditions indicate that the difference is significant (*P* < 0.05).

Studies on *DES1* and *OAS-A1* in Arabidopsis showed that cysteine is an important determinant of antioxidant capacity in the cytoplasm (Loìpez-Martiìn et al., 2008; Alvarez et al., 2010a), while *SCS* plays an important role in chloroplast redox (Bermúdez et al., 2010). In addition, cysteine and GSH are also associated with plant antioxidants. Therefore, we measured the superoxide anion (OFR) and hydrogen peroxide (H_2_O_2_) contents and the enzymatic activity of several antioxidant enzymes. Under normal growth conditions, although the H_2_O_2_ and OFR contents in the overexpression line were lower than those in the CK line, only the H_2_O_2_ content differed significantly (Figures 7A,B). The levels in both lines increased significantly after alkali stress, but the levels in the overexpression lines were significantly lower than those in the CK line. Thus, we speculated that the overexpression lines may be superior to the CK line in terms of the scavenging ability of reactive oxygen species (ROS).

Based on the above speculation, we also measured the activities of peroxidase (POD) and superoxide dismutase (SOD). The results showed that the activities of both enzymes were much higher in the overexpression lines than in the CK plants under alkali stress, and the activities of POD in the former were also significantly higher than those in the CK plants under normal growth conditions (Figures 7C,D). In conclusion, it was clear that the overexpression lines could reduce the accumulation of ROS by increasing the activity of antioxidant enzymes under alkali stress to enhance the alkali tolerance of the overexpression lines.

## Discussion

The CSase gene family is widespread in plants, and its members play an important role in cysteine synthesis, cyanide metabolism and other pathways. The synthesis of cysteine is the main function of the members of this gene family, and the CSase they encode is involved in the final step of cysteine synthesis. Cysteine is the first reduced sulfur donor organic molecule synthesized in plants and is involved in the synthesis of a variety of compounds involved in defense, redox, and other functions and occupies a central position in plant metabolism. Most studies on the classification and functional determination of the CSase gene family have focused on Arabidopsis (Barroso et al., 1995; Domínguez-Solís et al., 2004; Alvarez et al., 2010a; Álvarez et al., 2012a; Birke et al., 2012). However, studies on the identification, classification and related functions of CSase gene family members in specific species are lacking. For this reason, we conducted the present study on the MsCSase gene family in alfalfa.

The 39 *MsCSase* genes that were identified were unevenly distributed across only a few chromosomes. Although previous studies have also shown that *CSase* genes are unevenly distributed on only a few specific chromosomes, they have not shown that the genes are subject to fragment duplication or tandem duplication events (Yamaguchi et al., 2000; Akbudak et al., 2018; Liu et al., 2018, 2019). However, we identified two pairs of tandem repeat genes and one pair of gene-generating fragment duplication genes in alfalfa (Bennetzen et al., 2005; Zhu et al., 2014). These results suggested that tandem and fragment duplication events play a role in expansion of the alfalfa MsCSase gene family.

Based on phylogenetic analysis and previous Arabidopsis studies, we divided the alfalfa MsCSase gene family members (excluding *MsCSase25*, *MsCSase27*, and *MsCSase28*) into 5 subfamilies. The gene structures, motifs and domains of each subfamily member were somewhat similar. The results of the structural analysis support the reliability of the phylogenetic analysis. Differences in the physicochemical properties and structures of different subfamilies may result in differences in the activity of the enzymes or the function of the genes (Liszewska et al., 2007; Noda et al., 2016). The identified *MsCSase25*, *MsCSase27*, and *MsCSase28*, which do not belong to other subfamilies, although they have PLP-binding sites, still need to be verified whether they encode active cysteine synthases.

In terms of gene function validation, we successfully cloned the *CSase* gene (*MsCSase32*, *MsCSase34*) and produced *CSase*-overexpressing alfalfa. Stress tests showed that overexpression of *CSase* significantly improved alkali tolerance in alfalfa. Alkali stress signals induce the biosynthesis and accumulation of compatible osmotic solutes, including SSs and Pro, to improve tolerance. When the plants were subjected to alkali stress, compared with the CK plants, the overexpression lines accumulated more of these substances to reduce the intracellular osmotic potential, and the tolerance of the overexpression plants was improved by the accumulation of these substances.

CSase activity and the cysteine content increase under metal and salt stresses and that overexpression of *CSase* improves the antioxidant capacity and tolerance of plants (Domìnguez-Solìs et al., 2001; Youssefian et al., 2001; Domínguez-Solís et al., 2004; Fediuc et al., 2005; Pajuelo et al., 2007; Gotor et al., 2015). GSH content increases in overexpression lines under environmental stresses (Sabetta et al., 2017) and GSH plays important roles in scavenging ROS and transducing stress signals (Meyer, 2008; Zagorchev et al., 2013; Leng et al., 2015). In addition, redox- and ROS-dependent regulatory networks are important for photosynthesis in chloroplasts (Strand et al., 2015; Gütle et al., 2016). In the present study, we found that the increased resistance of the transgenic lines are due to the overexpression of the *CSase* gene leading to an increase in the content of antioxidants such as cysteine and downstream GSH, which in turn leads to enhanced antioxidant capacity. This was also evidenced by the increase in SOD and POD activities and the decrease in H_2_O_2_ and OFR contents in overexpression plants under alkali stress. Moreover, the enhanced antioxidant capacity of the transgenic lines may make their photosynthesis less affected by alkali stress and subsequently have higher SPAD values. The enhanced stress resistance resulted in good phenotypes of overexpression lines under alkali stress. In conclusion, CSase overexpression lines enhance plant tolerance by increasing the antioxidant capacity of plants. This conclusion is also consistent with the results of the previous group, which showed that *CSase* can respond to alkali stress and that increased cysteine content can improve the antioxidant capacity of the plants (Song et al., 2017, 2021).

Interestingly, numerous studies have shown that *CYS-C1* has dual functions in synthesizing cysteine and β-cyanoalanine (Ikegami et al., 1988; Watanabe et al., 2008; Marrero-Degro et al., 2010). However, *CYS-C1* currently synthesizes CAS only in mitochondria. The encoded *CSase* product (CAS) is localized in the mitochondria of Arabidopsis and is involved in the detoxification of HCN (Álvarez et al., 2012b). In the present study, this protein was localized in chloroplasts and enhanced the alkalinity tolerance of plants by synthesizing cysteine. From this, we hypothesized that *CSase* could have the ability to encode both CSase and CAS but selectively encodes one of the enzymes depending on the expression location. The above hypothesis needs to be further investigated. Whether *CSase* in alfalfa can also encode CAS also needs to be further investigated.

## Conclusion

In this study, we focused on 39 alfalfa MsCSase family members and classified them into 6 subfamilies first on the basis of the results of a phylogenetic analysis followed by a gene structure analysis, conserved domain characterization and a synteny analysis and on the basis of the high similarity in these aspects of members within the same subfamily. Subsequently, we cloned *CSase* and successfully overexpressed it in alfalfa. Evidence from both physiological experiments and the determination of the relative expression of downstream genes indicated that the overexpression lines can significantly improve alkali stress tolerance in alfalfa by increasing oxidative stress protection and the levels of osmoregulatory substances. These findings set the stage for the study of the CSase gene family. We will focus our future work on the associated metabolic pathways to further clarify the molecular mechanism of basal tolerance.

## Data Availability Statement

The original contributions presented in the study are included in the article/Supplementary Material, further inquiries can be directed to the corresponding author.

## Author Contributions

GC and YY designed the experiments. YY wrote the first draft of the article and GC revised it. All authors participated in the experiments and read and approved the final manuscript.

## Conflict of Interest

The authors declare that the research was conducted in the absence of any commercial or financial relationships that could be construed as a potential conflict of interest.

## Publisher’s Note

All claims expressed in this article are solely those of the authors and do not necessarily represent those of their affiliated organizations, or those of the publisher, the editors and the reviewers. Any product that may be evaluated in this article, or claim that may be made by its manufacturer, is not guaranteed or endorsed by the publisher.

## Footnotes

1
http://ncbi.nlm.nih.gov/

2
https://figshare.com/projects/whole_genome_sequencing_and_assembly_of_Medicago_sativa/66380

3
https://pfam.xfam.org/

4
http://meme-suite.org/tools/meme

5
https://www.ncbi.nlm.nih.gov/Structure/bwrpsb/bwrpsb.cgi
