# Supplementary material for: Genome-Wide Investigation of the Cysteine Synthase Gene Family Shows That Overexpression of CSase Confers Alkali Tolerance to Alfalfa (Medicago sativa L.)
Source: Front Plant Sci. 2022 Jan 4;12:792862. doi: 10.3389/fpls.2021.792862 (PMC8765340; doi:10.3389/fpls.2021.792862)
Supplement: Supplementary file 1 [file Data_Sheet_1.docx]

Supplementary Material

# Supplementary Figures and Tables

## Supplementary Figures


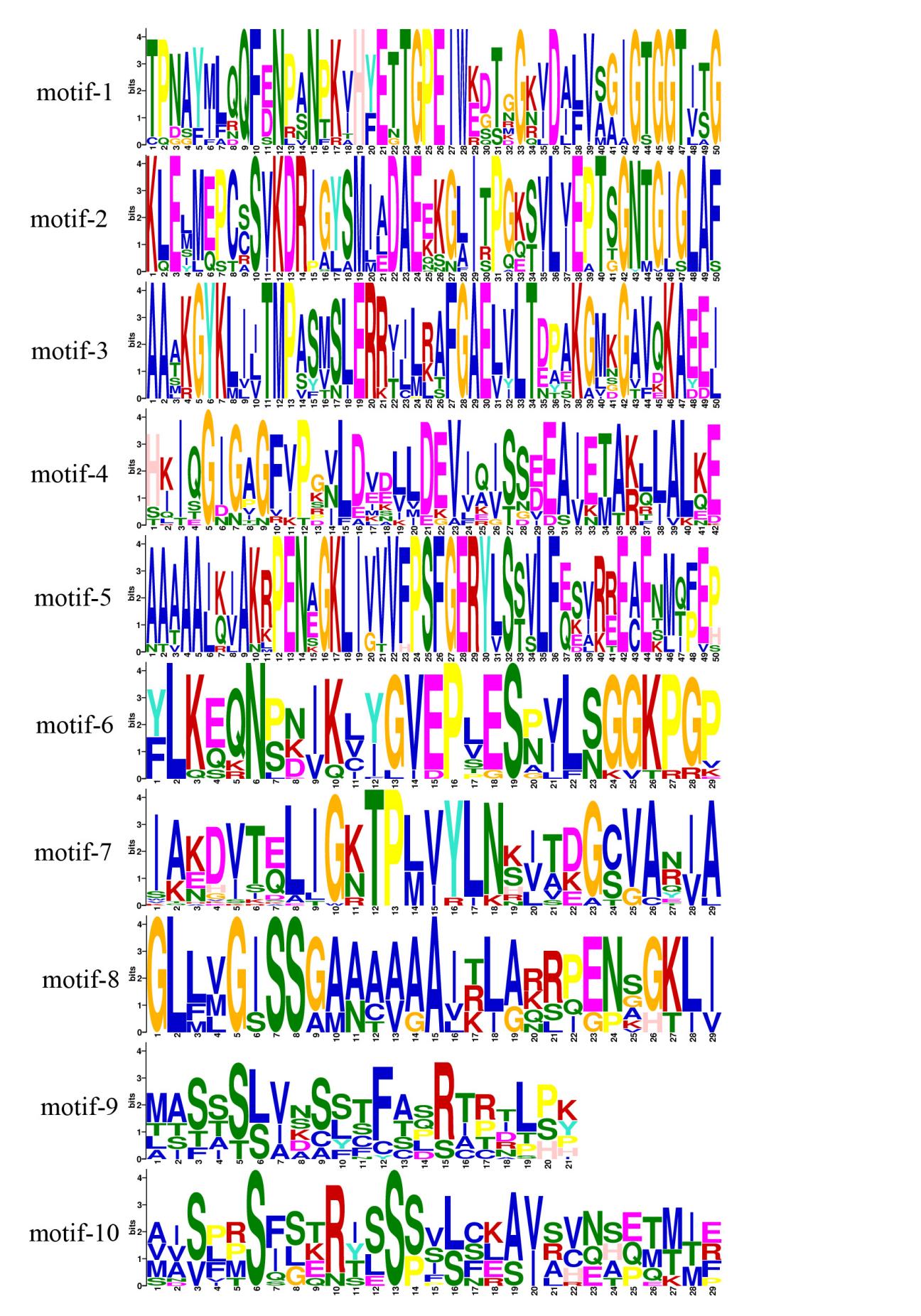


**Supplementary Figure 1. Sequence logo analysis of conserved motifs.**

**
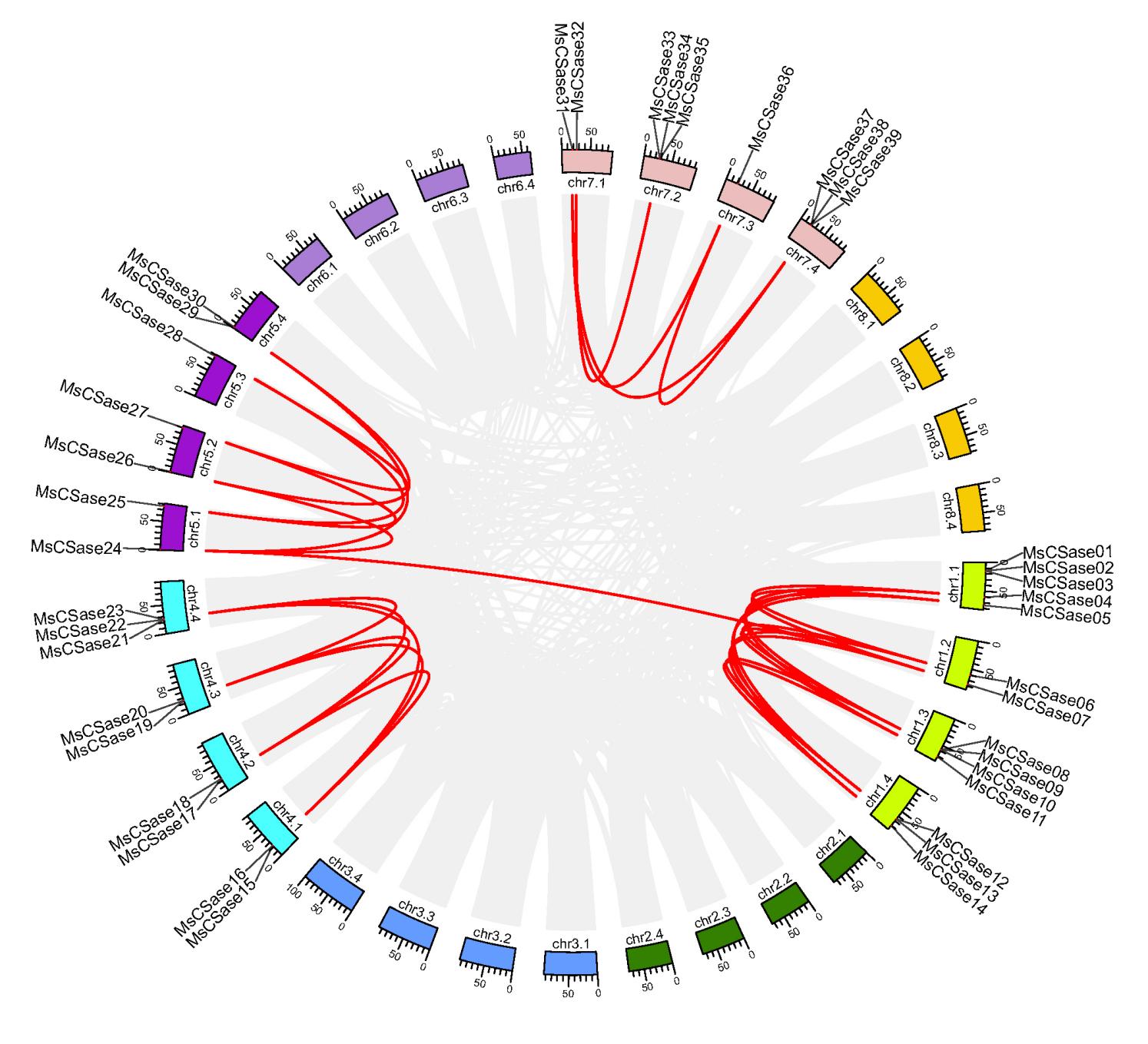
**

**Supplementary Figure 2. Synteny analysis of *MsCSase* genes in alfalfa.** Schematic representation of the chromosomal distribution and interchromosomal relationships of alfalfa *MsCSase* genes. The gray lines in the background show all gene pairs in alfalfa, while the red lines highlight the syntenic *MsCSase* gene pairs.

**Supplementary Figure 3. The amino acid sequence alignment results of *MsCSas32*, *MsCSase34* and *CSase* were compared.** The nucleotide sequence similarity between the three genes was 100%.

**Supplementary Figure 4. Nucleotide sequence alignment results of *MsCSas32*, *MsCSase34* and *CSase*.** The nucleotide sequence similarity of the three genes was 99.68%, while the nucleotide sequence similarity of *MsCSase34* and *CSase* was 99.91%.


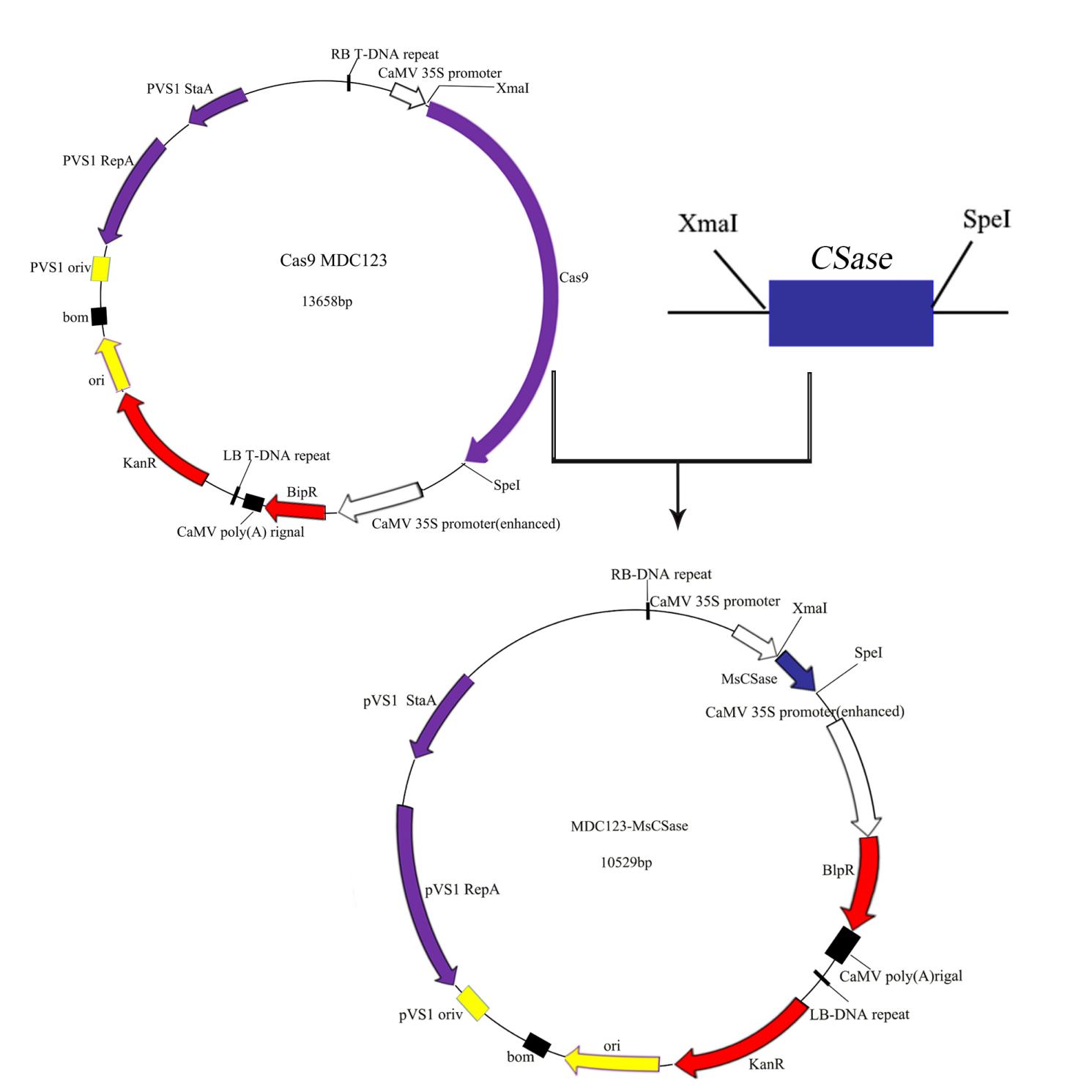


**Supplementary Figure 5. Schematic diagram of the construction of the alfalfa overexpression vector.** The blue rectangle represents the *CSase* target gene for this assay.


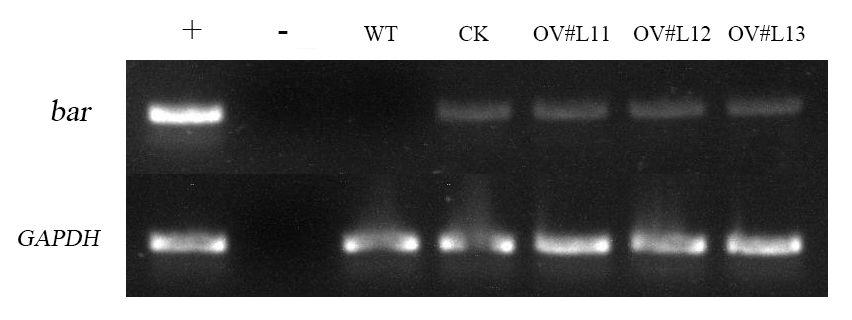


**Supplementary Figure 6. Identification of overexpression lines.** The upper and lower blots represent the PCR products of different cDNAs for the bar gene and reference gene, respectively. "+", "-", "WT", "CK", "OV#L11", "OV#L12" and "OV#L13" respectively represent the vector plasmid; negative control; wild-type plants; and *CSase* overexpression lines OV#L11, OV#L12 and OV#L13. b Relative expression of *CSase* genes in the CK lines and overexpression lines.

## Supplementary Tables

**Table S1. Information concerning the sequences of the primers used in this experiment**

| Primer name | Sequence (5' to 3') |
| --- | --- |
| *GAPDH* S | GGCTGCCATCAAGGAGGAAT |
| *GAPDH* AS | TCCAAGCTCAGCCTCATCAAG |
| CSase-GFP S | CGGGGGGACTCTTGACGAGCTCATGGCTTCTCTAATGAGTTTCC |
| CSase-GFP AS | CATGTCGACTCTAGAGGATCCATCGACCGAGACTGGCTG |
| *CSase* Real-time PCR S | ACAGTCGCAGCACTCAGATT |
| *CSase* Real-time PCR AS | CGAGACTGGCTGCATGTTCT |
| *CSase* S | ATGGCTTCTCTAATGAGTTTCCTC |
| *CSase* AS | TTAATCGACCGAGACTGGCTG |
| *bar* S | ATGGCTTCTCTAATGAGTTTCCTC |
| *bar* AS | TTAATCGACCGAGACTGGCTG |
| *Msγ-ECS* S | GGCCTAGCTTTGCAGCCTAT |
| *Msγ-ECS* AS | CATGCCTGTGCGGTCCTTAT |

**Table S2. List of basic information about the 39 *MsCSase* genes identified**

| **Gene name** | **Sequence ID** | **chromosome** | **Coordinate (5′-3′)** | **Protein** | | |
| --- | --- | --- | --- | --- | --- | --- |
|  |  |  |  | **Length(aa)** | **pI** | **MW(kDa)** |
| *MsCSase01* | MS.gene058739.t1 | chr1.1 | 14912055:14920821 | 585 | 5.53 | 61.72 |
| *MsCSase02* | MS.gene058737.t1 | chr1.1 | 14936950:14940270 | 305 | 5.81 | 32.45 |
| *MsCSase03* | MS.gene058735.t1 | chr1.1 | 14950904:14954213 | 308 | 6.04 | 32.44 |
| *MsCSase04* | MS.gene005160.t1 | chr1.1 | 60481524:60484877 | 325 | 5.47 | 34.45 |
| *MsCSase05* | MS.gene80045.t1 | chr1.1 | 74571697:74574245 | 325 | 5.92 | 34.17 |
| *MsCSase06* | MS.gene70799.t1 | chr1.2 | 60990654:60994011 | 325 | 5.47 | 34.45 |
| *MsCSase07* | MS.gene006455.t1 | chr1.2 | 77233468:77236064 | 325 | 5.92 | 34.14 |
| *MsCSase08* | MS.gene44030.t1 | chr1.3 | 57602017:57612014 | 325 | 5.35 | 34.42 |
| *MsCSase09* | MS.gene44032.t1 | chr1.3 | 57629551:57633459 | 321 | 6.06 | 34.45 |
| *MsCSase10* | MS.gene44033.t1 | chr1.3 | 57638602:57641315 | 323 | 5.17 | 34.59 |
| *MsCSase11* | MS.gene33576.t1 | chr1.3 | 71080161:71082678 | 325 | 5.92 | 34.16 |
| *MsCSase12* | MS.gene69913.t1 | chr1.4 | 66085492:66088859 | 325 | 5.46 | 34.43 |
| *MsCSase13* | MS.gene69911.t1 | chr1.4 | 66109529:66112570 | 318 | 5.47 | 33.93 |
| *MsCSase14* | MS.gene26887.t1 | chr1.4 | 80218332:80220880 | 325 | 5.92 | 34.17 |
| *MsCSase15* | MS.gene26579.t1 | chr4.1 | 22699634:22705065 | 383 | 6.07 | 40.66 |
| *MsCSase16* | MS.gene26578.t1 | chr4.1 | 22706753:22709425 | 364 | 8.6 | 39.17 |
| *MsCSase17* | MS.gene39577.t1 | chr4.2 | 23906372:23911803 | 383 | 6.07 | 40.66 |
| *MsCSase18* | MS.gene39578.t1 | chr4.2 | 23916043:23918724 | 364 | 8.6 | 39.16 |
| *MsCSase19* | MS.gene47527.t1 | chr4.3 | 25712711:25718209 | 383 | 6.07 | 40.66 |
| *MsCSase20* | MS.gene47526.t1 | chr4.3 | 25720781:25723479 | 366 | 8.09 | 39.33 |
| *MsCSase21* | MS.gene008717.t1 | chr4.4 | 25218066:25223498 | 383 | 6.07 | 40.66 |
| *MsCSase22* | MS.gene02466.t1 | chr4.4 | 25232287:25237719 | 383 | 6.07 | 40.66 |
| *MsCSase23* | MS.gene02467.t1 | chr4.4 | 25239373:25242045 | 364 | 8.6 | 39.16 |
| *MsCSase24* | MS.gene92293.t1 | chr5.1 | 1142529:1146138 | 325 | 5.69 | 34.27 |
| *MsCSase25* | MS.gene37942.t1 | chr5.1 | 76422790:76426337 | 422 | 8.38 | 45.08 |
| *MsCSase26* | MS.gene21844.t1 | chr5.2 | 1139958:1143581 | 325 | 5.69 | 34.25 |
| *MsCSase27* | MS.gene81336.t1 | chr5.2 | 80435226:80438918 | 422 | 8.37 | 45.13 |
| *MsCSase28* | MS.gene020786.t1 | chr5.3 | 77416155:77419703 | 422 | 8.38 | 45.08 |
| *MsCSase29* | MS.gene000153.t1 | chr5.4 | 2041881:2045490 | 325 | 5.69 | 34.27 |
| *MsCSase30* | MS.gene024871.t1 | chr5.4 | 2132014:2135623 | 325 | 5.69 | 34.27 |
| *MsCSase31* | MS.gene013867.t1 | chr7.1 | 18478098:18485903 | 661 | 8.92 | 73.6 |
| *MsCSase32* | MS.gene023918.t1 | chr7.1 | 25756449:25759466 | 379 | 8.28 | 40.94 |
| *MsCSase33* | MS.gene054826.t1 | chr7.2 | 27639266:27642300 | 407 | 9.01 | 44.17 |
| *MsCSase34* | MS.gene058651.t1 | chr7.2 | 27729291:27732295 | 379 | 8.28 | 40.94 |
| *MsCSase35* | MS.gene52446.t1 | chr7.2 | 27760654:27763686 | 394 | 9.08 | 42.67 |
| *MsCSase36* | MS.gene41829.t1 | chr7.3 | 21739441:21746649 | 800 | 9 | 88.96 |
| *MsCSase37* | MS.gene042525.t1 | chr7.4 | 20736283:20739477 | 359 | 8.2 | 38.53 |
| *MsCSase38* | MS.gene042523.t1 | chr7.4 | 20751570:20754304 | 342 | 6.77 | 36.45 |
| *MsCSase39* | MS.gene83857.t1 | chr7.4 | 20785170:20787904 | 342 | 6.77 | 36.45 |

**Table S3. List of sequence information concerning the coding DNA and protein sequences of the 39 *MsCSase* genes**

| **Gene Name** | **Gene ID** | **Sequence Type** | **Sequences** |
| --- | --- | --- | --- |
| *MsCSase01* | MS.gene058739.t1 | CDS | TTAATAGGAAACACACCATTGGTATATTTAAACAACATTACAGAAGGGTGTGTAGCTCGTATTGCTGCTAAGCTAGAATACTTGCAATCCTGCTGCAGTGTTAAAGATAGGATATCGTTGAGCATGATTGAAGATGCCGAGAGTAAAGGCCTCATTACACCTGGAAAGACTGTTCTTGTTGAGCCAACAAGTGGTAACACCGTCATAGGATTGGCGTCGATAGCAGCAATGAGGGGCTATAAACTTTTAGTGACTATGCCAGCTTATGTGAGTCTTGAAAGAAAAATTATACTGCGAGCTTTCGGAGCCGAGGTATATTTGACAGACCCTGCCAAAGGAATTGATGGTGTTTTTCAGAAAGCAGACGAGCTATTAGCGAAGACGCCTAATAGTTATATGCTCAATCAGTTTGAAAACTCTGCCAATCCAAAGATTCATTACGAGACCACTGGCCCTGAGATTTGGAAAGACTCTGGAGGGAGAGTTGATGCTTTGGTTGCAGGGATAGGAACTGGGGGAACAGTAACAGGTGTTGGGAAGTTCCTCAAGGAGAAAAACCCAGAGATCAAGGTGTATGGTGTAGAACCATCTGAAAGTGCAGTTCTGAATGGAGGAAAGCCAGGCAAACACCTAATCCAAGGGATCGGTGCTGGTGTCGTTCCCCCTATCCTGGAAGTTGATCTACTAGATGAAGTTATTGAGGTTTCAAGTGAAGAAGCTATAAAAACTGCTAAGCTGCTTGCCTTGAAAGAAGGTTTACTGATGGGAATTTCATCAGGTGCTGCAACAGCAGCTGCAATAAAATTAGGGAAGAGACCAGAAAATGCAGGAAAGCTCATTGTTTCTGGAACAGTTGACCTCAAGCCGTTAACTGAAGAAGTCAGGGATCAACTTGAGTTTGGAACACTTCTCTGTGGTCCTAGTTCGAGCACCACCTTGAATCTGGTGTTAAGTTACTGCGGGGTTGGAGTCCGGATAGCATTGAGCATGATTGAAGATGCTGAGAATAAAGGTCTCATTACTCCGGGAAAGAGTGTTCTTGTTGAGCCAACAAGTGGTAACACCGGCATAGGATTGGCGTCGATAGCAGCAATGAGGGGCTATAAACTTTTAGTGTCTTTGCCATTTTATGTGAGTCTTGAGAGAAGAATTCTACTCAGAGCTTTTGGAGCTGAGGTATATCTAACAGATCCTGCCAAAGGAATAAAAGGTGTTTTTGAGAAAGCGGAAGAGCTATTAGCGAAGACGCCTAATAGTTATATGTTCAATCAATTTGAAAATCCTGCCAATCCAAAGATTCATTATGAGACCACTGGCCCTGAGATTTGGAGAGACTCTGGAGGGAAAATTGATGCTTTGGTTGCAGGGATAGGAACTGGAGGAACAGTAACAGGTGTGTATGGTGTAGAACCAACTGAAAGTCCAGTTCTGAATGGAGGAAAGTCAGGCAATCATCGAATCCAAGGGATCGGTGCTGGTATCATTCCTCCTGTTCTGGAAGTTGATCTTCTAGATGAAGTTATTCAGGTTTCAAGTGAAGAAGCTATAAAAACTGCTAAGCTGCTTGCCTTGAAAGAAGGTTTACTGATGGGAATTTCATCAGGAGCTGCAGCTGCTGCTGCAATTAAACTAGGGAAGAGACCAGAAAATGTAGGAAAGCTCATTGTTGTGGTTTTTCCTAGTTCTGGAGAGCGTTACTTATCTTCAATACTTTTTGAGTCCATCAGGCATGAAGCTGAACAAATGACATTTGATTGA |
|  |  | Protein | LIGNTPLVYLNNITEGCVARIAAKLEYLQSCCSVKDRISLSMIEDAESKGLITPGKTVLVEPTSGNTVIGLASIAAMRGYKLLVTMPAYVSLERKIILRAFGAEVYLTDPAKGIDGVFQKADELLAKTPNSYMLNQFENSANPKIHYETTGPEIWKDSGGRVDALVAGIGTGGTVTGVGKFLKEKNPEIKVYGVEPSESAVLNGGKPGKHLIQGIGAGVVPPILEVDLLDEVIEVSSEEAIKTAKLLALKEGLLMGISSGAATAAAIKLGKRPENAGKLIVSGTVDLKPLTEEVRDQLEFGTLLCGPSSSTTLNLVLSYCGVGVRIALSMIEDAENKGLITPGKSVLVEPTSGNTGIGLASIAAMRGYKLLVSLPFYVSLERRILLRAFGAEVYLTDPAKGIKGVFEKAEELLAKTPNSYMFNQFENPANPKIHYETTGPEIWRDSGGKIDALVAGIGTGGTVTGVYGVEPTESPVLNGGKSGNHRIQGIGAGIIPPVLEVDLLDEVIQVSSEEAIKTAKLLALKEGLLMGISSGAAAAAAIKLGKRPENVGKLIVVVFPSSGERYLSSILFESIRHEAEQMTFD* |
| *MsCSase02* | MS.gene058737.t1 | CDS | ATGTCACGGAATATATCTCAACCATGTTGTGGATGGTTTGTGCTTGCACAAATTGCGGCAAAGCTAGAAATGATGGAACCTTGCTCTAGTGTCAAAGACAGGATAGGATATAGCATGATTGCAGATGCTGAGGAGAAAGGACTCATTAGACCGCAAGAAAGTGTCCTCATTGAGCCTACTAGTGGATACACTGGTATTGGTTTGGCATTCATGGCAGCTGCTAAGGGCTATAAACTTATTATAACCATGCCTGCTTCAATGAGTCTTGAGAGAAGAACCATTCTTCGCGCTTTTGGTGCCGAGTTAGTTCTCACTGATCCAGCTAAAGGTATGAAAGGAGCTGTTCAGAAAGCTGAAGAGATATTAGCAAAGACTCCCAATGCTTACATTCTTCAGCAATTTGAAAATCCTGCAAACCCAAAGGTTCACTATGAAACTACTGGACCAGAGATCTGGAAAAGCTCTGGTGGGAGAGTTGATGCCCTTGTTTCTGGTATAGGAACCGGAGGTACGGTAACAGGTGCTGGAAAATATTTGAAAGAGCAAAACCCTGATATTAAGCTATATGGTGTGGAACCACTTGAAAGTCCAGTATTGTCTGGAGGGAAACCTGGCCCTCATAAGATCCAAGGTATTGGTGCTGGCTTCATTCCCGGTGTTCTGGATGTTGATTTGCTTGATGAAGTAATCCAAATATCCAGCGAAGAAGCTATTGAAACTGCTAAACTTCTTGCATTAAAAGAAGGTGGGATATCATCTGGTGCTGCCGCAGCCGCTGCAATTAAGATTGCAAAGAAGCCGGAAAATGCTGGAAAACTCATTGTTGTTGTTTTCCCGAGCTTTGGAGAGCGTTATCTATCATCTGTGCTCTTTGAATCAGTGAAGCGAGAAGCCGAAAACATGATCTTTGAGCATTGA |
|  |  | Protein | MSRNISQPCCGWFVLAQIAAKLEMMEPCSSVKDRIGYSMIADAEEKGLIRPQESVLIEPTSGYTGIGLAFMAAAKGYKLIITMPASMSLERRTILRAFGAELVLTDPAKGMKGAVQKAEEILAKTPNAYILQQFENPANPKVHYETTGPEIWKSSGGRVDALVSGIGTGGTVTGAGKYLKEQNPDIKLYGVEPLESPVLSGGKPGPHKIQGIGAGFIPGVLDVDLLDEVIQISSEEAIETAKLLALKEGGISSGAAAAAAIKIAKKPENAGKLIVVVFPSFGERYLSSVLFESVKREAENMIFEH* |
| *MsCSase03* | MS.gene058735.t1 | CDS | ATGGCGGAAGACAAGTCAACCATTGCTAAAGATGTCACGGAATTGATTGGTAAAACTCCACTAGTATATCTCAACCATGTTGTGGATGGTTGTGTTGCACAAATTGCGGCAAAGCTAGAAATGATGGAACCTTGCTCTAGTGTCAAAGACAGGATAGGATATAGCATGATTGCAGATGCTGAGGAGAAAGGACTCATTAGACCGCAAGAAAGTGTCCTCATTGAGCCTACTAGTGGAAACACTGGTATTGGTTTGGCATTCATGGCAGCTGCTAAGGGCTATAAACTTATTATAACCATGCCTGCTTCAATGAGTCTTGAGAGAAGAACCATTCTTCGCGCTTTTGGTGCCGAGTTAGTTCTCACTGATCCAGCTAAAGGTATGAAAGGAGCTGTTCAGAAAGCTGAAGAGATATTAGCAAAGACTCCCAATGCTTACATTCTTCAGCAATTTGAAAATCCTGCAAACCCAAAGGTTCACTATGAAACTACTGGACCAGAGATCTGGAAAAGCTCTGGTGGGAGAGTTGATGCCCTTGTTTCTGGTATAGGAACCGGAGGTACGGTAACAGGTGCTGGAAAATATTTGAAAGAGCAAAACCCTGATATTAAGCTATATGGTGTGGAACCACTTGAAAGTCCAGTATTGTCTGGAGGGAAACCTGGCCCTCATAAGATCCAAGGTATTGGTGCTGGCTTCATTCCCGGTGTTCTGGATGTTGATTTGCTTGATGAAGTAATCCAAATATCCAGCGAAGAAGCTATTGAAACTGCTAAACTTCTTGCATTAAAGAAGGTGGGGATATCATCTGGTGCTGCCGCAGCCGCTGCAATTAAGATTGCAAAGAAGCCGGAAAATGCTGGAAAACTCATTGTTGTTGTTTTCCCCGAGCTTTGGAGAGCGTTATCTATCATCTGTGCTCTTTGA |
|  |  | Protein | MAEDKSTIAKDVTELIGKTPLVYLNHVVDGCVAQIAAKLEMMEPCSSVKDRIGYSMIADAEEKGLIRPQESVLIEPTSGNTGIGLAFMAAAKGYKLIITMPASMSLERRTILRAFGAELVLTDPAKGMKGAVQKAEEILAKTPNAYILQQFENPANPKVHYETTGPEIWKSSGGRVDALVSGIGTGGTVTGAGKYLKEQNPDIKLYGVEPLESPVLSGGKPGPHKIQGIGAGFIPGVLDVDLLDEVIQISSEEAIETAKLLALKKVGISSGAAAAAAIKIAKKPENAGKLIVVVFPELWRALSIICAL* |
| *MsCSase04* | MS.gene005160.t1 | CDS | ATGGCGGAAGACAAGTCAACCATTGCTAAAGATGTCACGGAATTGATTGGTAAAACTCCACTAGTATATCTCAACCATGTTGTGGATGGTTGTGTTGCACAAATTGCGGCAAAGCTAGAAATGATGGAACCTTGCTCTAGTGTCAAAGACAGGATAGGATATAGCATGATTGCAGATGCTGAGGAGAAAGGACTCATTAGACCGCAAGAAAGTGTCCTCATTGAGCCTACTAGTGGAAACACTGGTATTGGTTTGGCATTCATGGCAGCTGCTAAGGGCTATAAACTTATTATAACCATGCCTGCTTCAATGAGTCTTGAGAGAAGAACCATTCTTCGCGCTTTTGGTGCCGAGTTAGTTCTCACTGATCCAGCTAAAGGTATGAAAGGAGCTGTTCAGAAAGCTGAAGAGATATTAGCAAAGACTCCCAATGCTTACATTCTTCAGCAATTTGAAAATCCTGCAAACCCAAAGGTTCACTATGAAACTACTGGACCAGAGATCTGGAAAAGCTCTGGTGGGAGAGTTGATGCCCTTGTTTCTGGTATAGGAACCGGAGGTACGGTAACAGGTGCTGGAAAATATTTGAAAGAGCAAAACCCTGATATTAAGCTATATGGTGTGGAACCACTTGAAAGTCCAGTATTGTCTGGAGGGAAACCTGGCCCTCATAAGATCCAAGGTATTGGTGCTGGCTTCATTCCCGGTGTTCTGGATGTTGATTTGCTTGATGAAGTAATCCAAATATCCAGCGAAGAAGCTATTGAAACTGCTAAACTTCTTGCATTAAAAGAAGGTTTGCTGGTGGGGATATCATCTGGTGCTGCCGCAGCCGCTGCAATTAAGATTGCAAAGAAGCCGGAAAATGCTGGAAAACTCATTGTTGTTGTTTTCCCGAGCTTTGGAGAGCGTTATCTATCATCTGTGCTCTTTGAATCAGTGAAGCGAGAAGCCGAAAACATGATCTTTGAGCATTGA |
|  |  | Protein | MAEDKSTIAKDVTELIGKTPLVYLNHVVDGCVAQIAAKLEMMEPCSSVKDRIGYSMIADAEEKGLIRPQESVLIEPTSGNTGIGLAFMAAAKGYKLIITMPASMSLERRTILRAFGAELVLTDPAKGMKGAVQKAEEILAKTPNAYILQQFENPANPKVHYETTGPEIWKSSGGRVDALVSGIGTGGTVTGAGKYLKEQNPDIKLYGVEPLESPVLSGGKPGPHKIQGIGAGFIPGVLDVDLLDEVIQISSEEAIETAKLLALKEGLLVGISSGAAAAAAIKIAKKPENAGKLIVVVFPSFGERYLSSVLFESVKREAENMIFEH* |
| *MsCSase05* | MS.gene80045.t1 | CDS | ATGGCAGTTGAAAGGTCTGGAATAGCCAAAGATATTACAGAATTGATCGGAAAAACCCCGTTAGTATATCTGAATAAAATCACGGATGGTTCTGTTGCTCGAGTTGCTGCCAAACTGGAATTAATGGAACCATGCTCTAGTGTAAAAGACAGGATTGGCTATAGTATGATTGCTGATGCAGAGGAGAAGGGACTAATTACGCCTGGCCAGAGTGTCCTTATTGAGCCAACAAGTGGCAATACGGGCATCGGATTGGCCTTCTTGGCAGCAGCCAAAGGCTACAAGCTTATCATTACAATGCCTGCTTCAATGAGTCTTGAAAGAAGAACCATTCTTCTCTCTTTTGGAGCTGAGTTGGTTCTCACGGATCCTGCAAAGGGAATGAAAGGGGCAGTTCAGAAAGCTGAAGAGATATTAGCCAAGACTCCAAATGCCTATATCCTTCAGCAATTTGAAAACCCTGCCAATCCAAAGGTGCATTATGAAACCACTGGTCCAGAGATATGGAAAGGCACAGGAGGGAAAGTTGATGCATTTGTTAGTGGTATAGGGACTGGTGGTACCATAACAGGTGTTGGGAAATATCTTAAAGAGCAGAATTCCAATATCAAGCTGTATGGAGTGGAGCCAGTTGAAAGTCCCGTTCTGTCCGGAGGAAAGCCTGGTCCACACAAGATTCAAGGGATTGGTGCTGGTTTTGTTCCCGGGGTATTGGATGTTAGTCTTGTCGACGAAGTAATTCAAATATCAAGTGATGAAGCAATAGAAACTGCAAAGCTTCTTGCCCTTAAAGAAGGCCTATTTGTTGGAATATCATCTGGAGCTGCCGCGGCAGCTGCCATTAAGATAGCAAAACGACCAGAAAATGCTGGAAAGCTTATTGTTGTTGTTTTCCCTAGCTTTGGTGAGAGGTACCTATCCTCTGTGCTGTTTGAATCGGTGAGACGGGAGGCTGAAAGCCTGACTTTTGAACCATGA |
|  |  | Protein | MAVERSGIAKDITELIGKTPLVYLNKITDGSVARVAAKLELMEPCSSVKDRIGYSMIADAEEKGLITPGQSVLIEPTSGNTGIGLAFLAAAKGYKLIITMPASMSLERRTILLSFGAELVLTDPAKGMKGAVQKAEEILAKTPNAYILQQFENPANPKVHYETTGPEIWKGTGGKVDAFVSGIGTGGTITGVGKYLKEQNSNIKLYGVEPVESPVLSGGKPGPHKIQGIGAGFVPGVLDVSLVDEVIQISSDEAIETAKLLALKEGLFVGISSGAAAAAAIKIAKRPENAGKLIVVVFPSFGERYLSSVLFESVRREAESLTFEP* |
| *MsCSase06* | MS.gene70799.t1 | CDS | ATGGCGGAAGACAAGTCAACCATTGCTAAAGATGTCACGGAATTGATTGGTAAAACTCCACTAGTATATCTCAACCATGTTGTGGATGGTTGTGTTGCACAAATTGCGGCAAAGCTAGAAATGATGGAACCTTGCTCTAGTGTCAAAGACAGGATAGGATATAGCATGATTGCAGATGCTGAGGAGAAAGGACTCATTAGACCGCAAGAAAGTGTCCTCATTGAGCCTACTAGTGGAAACACTGGTATTGGTTTGGCATTCATGGCAGCTGCTAAGGGCTATAAACTTATTATAACCATGCCTGCTTCAATGAGTCTTGAGAGAAGAACCATTCTTCGCGCTTTTGGTGCCGAGTTAGTTCTCACTGATCCAGCTAAAGGTATGAAAGGAGCTGTTCAGAAAGCTGAAGAGATATTAGCAAAGACTCCCAATGCTTACATTCTTCAGCAATTTGAAAATCCTGCAAACCCAAAGGTTCACTATGAAACTACTGGACCAGAGATCTGGAAAAGCTCTGGTGGGAGAGTTGATGCCCTTGTTTCTGGTATAGGAACCGGAGGTACGGTAACAGGTGCTGGAAAATATTTGAAAGAGCAAAACCCTGATATTAAGCTATATGGTGTGGAACCACTTGAAAGTCCAGTATTGTCTGGAGGGAAACCTGGCCCTCATAAGATCCAAGGTATTGGTGCTGGCTTCATTCCTGGTGTTCTGGATGTTGATTTGCTTGATGAAGTAATCCAAATATCCAGCGAAGAAGCTATTGAAACTGCTAAACTTCTTGCATTAAAAGAAGGTTTGCTGGTGGGGATATCATCTGGTGCTGCCGCAGCCGCTGCAATTAAGATTGCAAAGAAGCCGGAAAATGCTGGAAAACTCATTGTTGTTGTTTTCCCGAGCTTTGGAGAGCGTTATCTATCATCTGTGCTCTTTGAATCAGTGAAGCGAGAAGCCGAAAACATGATCTTTGAGCATTGA |
|  |  | Protein | MAEDKSTIAKDVTELIGKTPLVYLNHVVDGCVAQIAAKLEMMEPCSSVKDRIGYSMIADAEEKGLIRPQESVLIEPTSGNTGIGLAFMAAAKGYKLIITMPASMSLERRTILRAFGAELVLTDPAKGMKGAVQKAEEILAKTPNAYILQQFENPANPKVHYETTGPEIWKSSGGRVDALVSGIGTGGTVTGAGKYLKEQNPDIKLYGVEPLESPVLSGGKPGPHKIQGIGAGFIPGVLDVDLLDEVIQISSEEAIETAKLLALKEGLLVGISSGAAAAAAIKIAKKPENAGKLIVVVFPSFGERYLSSVLFESVKREAENMIFEH* |
| *MsCSase07* | MS.gene006455.t1 | CDS | ATGGCAGTTGAAAGGTCTGGAATTGCCAAAGATGTTACAGAATTGATCGGAAAAACCCCATTAGTATATCTGAATAAAATCACGGATGGTTCTGTTGCTCGAGTTGCTGCCAAACTGGAATTAATGGAACCATGCTCTAGTGTAAAAGACAGGATTGGCTATAGTATGATTGCTGATGCAGAGGAGAAGGGACTAATTACGCCTGGCCAGAGTGTCCTTATTGAGCCAACAAGTGGCAATACGGGCATCGGATTGGCCTTCTTGGCAGCAGCCAAAGGCTACAAGCTTATCATTACAATGCCTGCTTCAATGAGTCTTGAAAGAAGAACCATTCTTCTATCTTTTGGAGCTGAGTTGGTTCTGACAGATCCTGCAAAGGGAATGAAAGGGGCAGTTCAGAAAGCTGAAGAGATATTAGCCAAGACTCCAAATGCCTATATCCTTCAGCAATTTGAAAACCCTGCCAATCCAAAGGTGCATTATGAAACCACTGGTCCAGAGATATGGAAAGGCACAGGAGGGAAAGTTGATGCATTTGTTAGTGGTATAGGTACTGGTGGTACCATAACAGGTGTTGGGAAATATCTTAAAGAGCAAAATTCCAATATCAAGCTGTTTGGGGTGGAGCCAGTTGAAAGTCCTGTACTGTCCGGAGGAAAGCCTGGTCCACACAAGATTCAAGGGATTGGTGCTGGTTTTGTTCCTGGGGTATTGGACGTTAGTCTTGTCGACGAAGTAATTCAAATATCAAGTGATGAAGCAATAGAAACTGCAAAGCTTCTTGCCCTTAAAGAAGGCCTATTTGTTGGAATATCCTCTGGAGCTGCTGCAGCAGCTGCCATTAAGATAGCAAAAAGACCAGAAAATGCTGGAAAGCTTATTGTTGTTGTTTTCCCTAGCTTTGGTGAGAGGTACCTATCTTCTGTGCTGTTTGAATCGGTGAGACGGGAGGCTGAAAGCCTGACTTTTGAACCATGA |
|  |  | Protein | MAVERSGIAKDVTELIGKTPLVYLNKITDGSVARVAAKLELMEPCSSVKDRIGYSMIADAEEKGLITPGQSVLIEPTSGNTGIGLAFLAAAKGYKLIITMPASMSLERRTILLSFGAELVLTDPAKGMKGAVQKAEEILAKTPNAYILQQFENPANPKVHYETTGPEIWKGTGGKVDAFVSGIGTGGTITGVGKYLKEQNSNIKLFGVEPVESPVLSGGKPGPHKIQGIGAGFVPGVLDVSLVDEVIQISSDEAIETAKLLALKEGLFVGISSGAAAAAAIKIAKRPENAGKLIVVVFPSFGERYLSSVLFESVRREAESLTFEP* |
| *MsCSase08* | MS.gene44030.t1 | CDS | ATGGCGGAAGACAAGTCAACCATTGCTAAAGATGTCACGGAATTGATTGGTAAAACTCCACTAGTATATCTCAACCATGTTGTGGATGGTTGTGTTGCACAAATTGCGGCAAAGCTAGAAATGATGGAACCTTGCTCTAGTGTCAAAGACAGGATAGGATATAGCATGATTGCAGATGCTGAGGAGAAAGGACTCATTAGACCGCAAGAAAGTGTCCTCATTGAGCCTACTAGTGGAAACACTGGTATTGGTTTGGCATTCATGGCAGCTGCTAAGGGCTATAAACTTATTATAACCATGCCTGCTTCAATGAGTCTTGAGAGAAGAACCATTCTTCGCGCTTTTGGTGCCGAGTTAGTTCTCACTGATCCAGCTAAAGGTATGAAAGGAGCTGTTCAGAAAGCTGAAGAGATATTAGCAAAGACTCCCAATGCTTACATTCTTCAGCAATTTGAAAATCCTGCAAACCCAAAGGTTCACTATGAAACTACTGGACCAGAGATCTGGAAAAGCTCTGGTGGGAAAGTTGATGCCCTTGTTTCTGGTATAGGAACCGGAGGTACGGTAACAGGTGCTGGAAAATATTTGAAAGAGCAAAACCCTGATATTAAGCTATATGGTGTGGAACCACTTGAAAGTCCAGTATTGTCTGGAGGGAAACCTGGCCCTCATAAGATCCAAGGTATTGGTGCTGGCTTCATTCCTGGTGTTCTGGATGTTGATTTGCTTGATGAAGTAATCCAAATATCCAGCGAAGAAGCTATTGAAACTGCTAAACTTCTTGCATTAAAAGAAGGTTTGCTGGTGGGGATATCATCTGGTGCTGCCGCAGCCGCTGCAATTAAGATTGCAAAGATGCCGGAAAATGCTGGAAAACTCATTGTTGTTGTTTTCCCGAGCTTTGGAGAGCGTTATCTATCATCTGTGCTCTTTGAATCAGTGAAGCGAGAAGCCGAAAACATGATCTTTGAGCATTGA |
|  |  | Protein | MAEDKSTIAKDVTELIGKTPLVYLNHVVDGCVAQIAAKLEMMEPCSSVKDRIGYSMIADAEEKGLIRPQESVLIEPTSGNTGIGLAFMAAAKGYKLIITMPASMSLERRTILRAFGAELVLTDPAKGMKGAVQKAEEILAKTPNAYILQQFENPANPKVHYETTGPEIWKSSGGKVDALVSGIGTGGTVTGAGKYLKEQNPDIKLYGVEPLESPVLSGGKPGPHKIQGIGAGFIPGVLDVDLLDEVIQISSEEAIETAKLLALKEGLLVGISSGAAAAAAIKIAKMPENAGKLIVVVFPSFGERYLSSVLFESVKREAENMIFEH* |
| *MsCSase09* | MS.gene44032.t1 | CDS | ATGGGTAAGGTTGTTAAGAGGAAGAAGAAGTCAGAAACATGGAACACACCATTGGTATATCTAAACAACATTACAGAAGGGTGTGTAGCTCGTATTGCTGCTAAGCTTGAATACTTGCAATCTTGCTGCAGTGTCAAAGATAGGATAGCATTGAGCATGATTGAAGATGCCGAAAATAATGGCTTCATTACTCCTGGAAAGTCTGTTCTTGTTGAGGCTACTAGTGGCAACACCGGCATAGGATTGGCATCGATCGCAGCACTGAGAGGCTATAAACTTTTAGTAACCATGCCATCTTTTGTGAGTCTTGAGAGAAAAATTATACTGCGCGCTTTTGGAGCCGAGGTATATCTGACAGACCCTGCCAAAGGAGTTGATGGAGTTTTTGAAAAGGCAGACGAGCTATTAGCAAAGACACCTAATAGCTATATGCTCAATCAATTTGAAAATCCTGTCAATCCAAAGACTCATTATGAGACCACTGGCCCTGAAATTTGGAGAGACACTGGAGGGAGAATTGATGCTTTGGTTGCAGGGATAGGAACTGGGGGAACAATAACAGGTGCTGGGAAGTTCCTCAAGGAGAGAAACTTAGATATCAAGGTGTATGGTGTAGAACCAACTGAAAGTGCAATTCTGAATGGAGGAAAGCCAGGCAAACACCTAATCCAAGGGATCGGTACTGGTGTCGTTCCCCCTGTCCTGGATCTTGATCTCCTAGATGAAGTTATTCAGGTTTCGAGTGAAGAAGCTATAGAAACTGCTAAGCTGCTTGCTTTGAAAGAAGGTTTACTGATGGGAATTTCATCAGGAGCTGCAGCAGCAGCAGCAATAAAACTAGGGAAGAGACAAGAAAATGCAGGAAAGCTCATTGTTGTGGTTTTTCCTAGTTTTGGAGAGCGTTACTTATCTTCAATACTTTTTGAGTCCATCAGGCATGAAGCTGAACAAATGATATTTGATTGA |
|  |  | Protein | MGKVVKRKKKSETWNTPLVYLNNITEGCVARIAAKLEYLQSCCSVKDRIALSMIEDAENNGFITPGKSVLVEATSGNTGIGLASIAALRGYKLLVTMPSFVSLERKIILRAFGAEVYLTDPAKGVDGVFEKADELLAKTPNSYMLNQFENPVNPKTHYETTGPEIWRDTGGRIDALVAGIGTGGTITGAGKFLKERNLDIKVYGVEPTESAILNGGKPGKHLIQGIGTGVVPPVLDLDLLDEVIQVSSEEAIETAKLLALKEGLLMGISSGAAAAAAIKLGKRQENAGKLIVVVFPSFGERYLSSILFESIRHEAEQMIFD* |
| *MsCSase10* | MS.gene44033.t1 | CDS | ATGGAGCAACACTTTGCAATTAAGAAGGATGTCACTCAATTAATAGGGAACACACCATTGGTATATTTAAACAACATTACAGAAGGGTGTGTAGCTCGTATTGCTGCTAAGCTAGAATACTTGCAATCCTGCTGCAGTATCAAAGATAGGATAGCATTGAGCATGATTGAAGATGCCGAAAATAAAGGTCTCATTACTCCTGCAAAGACTGTTCTTGTTGAGGCTACGGGTGGCAACACCGGCATAGGATTGGCATCGATCGCAGCACTGAGAGGCTATAAACTTTTAGTAACTATGCCATCTTTTGTGAGTCTTGAGAGAAAAATTATACCGCGAGCTTTCGGAGCCGAGGTATATCTGACAGACCCTGCCAAAGGAGTTGATGGAGTTTTTGAAAAGGCAGACGAGCTATTAGCAAAGACACCTAATAGCTATATGCTCAATCAATTTGAAAATCCTGTCAATCCAAAGACTCATTATGAGACCACTGGCCCTGAGATTTGGAGAGACACTGGAGGGAGAATTGATGCTTTGGTTGCTGGGATAGGAACTGGGGGAACAATAACAGGTGCTGGGAAGTTCCTCAAGGAGAGAAACTTAGATATCAAGGTGTATGGTGTAGAACCAACTGAAAGTGCAATTTTGAATGGAGGAAAGCCAGGCGAACATCTAATCCAAGGTATCGGTAATGGTGTCGTTCCCCCTGTCCTGGATCTTGATCTCCTAGATGAAGTTATTCAGGTTTCAAGTGAAGAAGCTATAGAAACTGCTAAGCTGCTTGCTTTGAAAGAAGGTTTACTGATGGGAATTTCATCAGGAGCTGCAGCAGCAGCTGCAATAAAACTAGGGAAGAGACAAGAAAATGCAGGAAAGCTCATTGTTGTGGTTTTTCCTAGTTTTGGAGAGCGTTACTTATCTTCACCACTTTTTGAATCCATCAGGCGTGAAGCTGAAGAAATGACATTTGACTGA |
|  |  | Protein | MEQHFAIKKDVTQLIGNTPLVYLNNITEGCVARIAAKLEYLQSCCSIKDRIALSMIEDAENKGLITPAKTVLVEATGGNTGIGLASIAALRGYKLLVTMPSFVSLERKIIPRAFGAEVYLTDPAKGVDGVFEKADELLAKTPNSYMLNQFENPVNPKTHYETTGPEIWRDTGGRIDALVAGIGTGGTITGAGKFLKERNLDIKVYGVEPTESAILNGGKPGEHLIQGIGNGVVPPVLDLDLLDEVIQVSSEEAIETAKLLALKEGLLMGISSGAAAAAAIKLGKRQENAGKLIVVVFPSFGERYLSSPLFESIRREAEEMTFD* |
| *MsCSase11* | MS.gene33576.t1 | CDS | ATGGCAGTTGAAAGGTCTGGAATTGCCAAAGATGTTACAGAATTGATCGGAAAAACCCCATTAGTATATCTGAATAAAATCACGGATGGTTCTGTTGCTCGAGTTGCTGCCAAACTGGAATTAATGGAACCATGCTCTAGTGTAAAAGACAGGATTGGCTATAGTATGATTGCTGATGCAGAGGAGAAGGGACTAATTACGCCTGGCCAGAGTGTCCTTATTGAGCCAACAAGTGGCAATACGGGCATCGGATTGGCCTTCTTGGCAGCAGCCAAAGGCTACAAGCTTATCATTACAATGCCTGCTTCAATGAGTCTTGAAAGAAGAACCATTCTTCTATCTTTTGGAGCTGAGTTGGTTCTGACAGATCCTGCAAAGGGAATGAAAGGGGCAGTTCAGAAAGCTGAAGAGATATTAGCCAAGACTCCAAATGCCTATATCCTTCAGCAATTTGAAAACCCTGCCAATCCAAAGGTGCATTATGAAACCACTGGTCCAGAGATATGGAAAGGCACAGGAGGGAAAGTTGATGCATTTGTTAGTGGTATAGGTACTGGTGGTACCATAACAGGTGTTGGGAAATATCTTAAAGAGCAGAATTCCAATATCAAGCTGTATGGGGTGGAGCCAGTTGAAAGTCCTGTACTGTCCGGAGGAAAGCCTGGTCCACACAAGATTCAAGGGATTGGTGCTGGTTTTGTTCCCGGGGTATTGGACGTTAGTCTTGTCGACGAAGTAATTCAAATATCAAGTGATGAAGCAATAGAAACTGCAAAGCTTCTTGCCCTTAAAGAAGGCCTATTTGTTGGAATATCATCTGGAGCTGCTGCGGCAGCTGCCATTAAGATAGCAAAAAGACCAGAAAATGCTGGAAAGCTTATTGTTGTTGTTTTCCCTAGCTTTGGTGAGAGGTACCTATCCTCTGTGCTGTTTGAATCGGTGAGACGGGAGGCTGAAAGCCTGACTTTTGAACCATGA |
|  |  | Protein | MAVERSGIAKDVTELIGKTPLVYLNKITDGSVARVAAKLELMEPCSSVKDRIGYSMIADAEEKGLITPGQSVLIEPTSGNTGIGLAFLAAAKGYKLIITMPASMSLERRTILLSFGAELVLTDPAKGMKGAVQKAEEILAKTPNAYILQQFENPANPKVHYETTGPEIWKGTGGKVDAFVSGIGTGGTITGVGKYLKEQNSNIKLYGVEPVESPVLSGGKPGPHKIQGIGAGFVPGVLDVSLVDEVIQISSDEAIETAKLLALKEGLFVGISSGAAAAAAIKIAKRPENAGKLIVVVFPSFGERYLSSVLFESVRREAESLTFEP* |
| *MsCSase12* | MS.gene69913.t1 | CDS | ATGGCGGATGACAAGTTAACCATTGCTAAAGATGTCACTGAATTGATTGGTAAAACTCCACTGGTATATCTCAATCATGTCGTGGATGGTTGTGTTGCACAAATTGCTGCAAAGCTAGAAATGATGGAACCTTGCTCTAGTGTCAAAGACAGGATAGGATATAGCATGATTGCAGATGCTGAGGAGAAAGGACTCATTAGACCGCAAGAAAGTGTCCTCATTGAGCCTACTAGTGGAAACACTGGTATTGGTTTGGCATTCATGGCAGCTGCTAAGGGCTATAAACTTATTATAACCATGCCTGCTTCAATGAGTCTTGAGAGAAGAACCATTCTTCGCGCTTTTGGTGCCGAGTTAGTTCTCACTGATCCAGCTAAAGGTATGAAAGGAGCTGTTCAGAAAGCTGAAGAGATATTAGCAAAGACTCCCAATGCTTACATTCTTCAGCAATTTGAAAATCCTGCAAACCCAAAGGTTCACTATGAAACTACTGGACCAGAGATCTGGAAAAGCTCTGGTGGGAAAGTTGATGCCCTTGTTTCTGGTATAGGAACCGGAGGTACGGTAACAGGTGCTGGAAAATATTTGAAAGAGCAAAACCCTGATATTAAGCTATATGGTGTGGAACCACTTGAAAGTCCAGTATTGTCTGGAGGGAAACCTGGCCCTCATAAGATCCAAGGTATTGGTGCTGGCTTCATTCCTGGTGTTCTGGATGTTGATTTGCTTGATGAAGTAATCCAAATATCCAGCGAAGAAGCTATTGAAACTGCTAAACTTCTTGCATTAAAAGAAGGTTTGCTGGTGGGGATATCATCTGGTGCTGCCGCAGCCGCTGCAATTAAGATTGCAAAGAAGCCGGAAAATGCTGGAAAACTCATTGTTGTTGTTTTCCCGAGCTTTGGAGAGCGTTATCTATCATCTGTGCTCTTTGAATCAGTGAAGCGAGAAGCCGAAAACATGATCTTTGAGCATTGA |
|  |  | Protein | MADDKLTIAKDVTELIGKTPLVYLNHVVDGCVAQIAAKLEMMEPCSSVKDRIGYSMIADAEEKGLIRPQESVLIEPTSGNTGIGLAFMAAAKGYKLIITMPASMSLERRTILRAFGAELVLTDPAKGMKGAVQKAEEILAKTPNAYILQQFENPANPKVHYETTGPEIWKSSGGKVDALVSGIGTGGTVTGAGKYLKEQNPDIKLYGVEPLESPVLSGGKPGPHKIQGIGAGFIPGVLDVDLLDEVIQISSEEAIETAKLLALKEGLLVGISSGAAAAAAIKIAKKPENAGKLIVVVFPSFGERYLSSVLFESVKREAENMIFEH* |
| *MsCSase13* | MS.gene69911.t1 | CDS | ATGAAAACAAAAGAATCAAAAGCATTAACAGGGAACACACCATTGGTATATCTAAACAACATTACAGAAGGGTGTGTAGCTCGTATTGCTGCTAAGCTTGAATACTTGCAATCTTGCTGCAGTGTCAAAGATAGGATAGCATTGAGCATGATTGAAGATGCCGAAAATAATGGCTTCATTACTCCTGGAAAGTCTGTTCTTGTTGAGGCTACTAGTGGCAACACCGGCATAGGATTGGCATCGATCGCAGCACTGAGAGGCTATAAACTTTTAGTAACCATGCCATCTTTTGTGAGTCTTGAGAGAAAAATTATACTGCGCGCTTTTGGAGCCGAGGTATATCTGACAGACCCTGCCAAAGGAGTTGATGGAGTTTTTGAAAAGGCAGACGAGCTATTAGCAAAGACACCTAATAGCTATATGCTCAATCAATTTGAAAATCCTGTCAATCCAAAGACTCATTATGAGACCACTGGCCCTGAAATTTGGAGAGACACTGGAGGGAGAATTGATGCTTTGGTTGCAGGGATAGGAACTGGGGGAACAATAACAGGTGCTGGGAAGTTCCTCAAGGAGAGAAACTTAGATATCAAGGTGTATGGTGTAGAACCAACTGAAAGTGCAATTTTGAATGGAGGAAAGCCAGGCAAACACCTAATCCAAGGGATCGGTACTGGTGTCGTTCCCCCTGTCCTGGATCTTGATCTCCTAGATGAAGTTATTCAGGTTTCAAGTGAAGAAGCTATAGAAACTGCTAAGCTGCTTGCTTTGAAAGAAGGTTTACTGATGGGAATTTCATCAGGAGCTGCAGCAGCAGCAGCAATAAAACTAGGGAAGAGACAAGAAAATGCAGGAAAGCTCATTGTTGTGGTTTTTCCTAGTTTTGGAGAGCGTTACTTATCTTCAATACTTTTTGAGTCCATCAGGCATGAAGCTGAACAAATGACATTTGATTGA |
|  |  | Protein | MKTKESKALTGNTPLVYLNNITEGCVARIAAKLEYLQSCCSVKDRIALSMIEDAENNGFITPGKSVLVEATSGNTGIGLASIAALRGYKLLVTMPSFVSLERKIILRAFGAEVYLTDPAKGVDGVFEKADELLAKTPNSYMLNQFENPVNPKTHYETTGPEIWRDTGGRIDALVAGIGTGGTITGAGKFLKERNLDIKVYGVEPTESAILNGGKPGKHLIQGIGTGVVPPVLDLDLLDEVIQVSSEEAIETAKLLALKEGLLMGISSGAAAAAAIKLGKRQENAGKLIVVVFPSFGERYLSSILFESIRHEAEQMTFD* |
| *MsCSase14* | MS.gene26887.t1 | CDS | ATGGCAGTTGAAAGGTCTGGAATAGCCAAAGATATTACAGAATTGATCGGAAAAACCCCGTTAGTATATCTGAATAAAATCACGGATGGTTCTGTTGCTCGAGTTGCTGCCAAACTGGAATTAATGGAACCATGCTCTAGTGTAAAAGACAGGATTGGCTATAGTATGATTGCTGATGCAGAGGAGAAGGGACTAATTACGCCTGGCCAGAGTGTCCTTATTGAGCCAACAAGTGGCAATACGGGCATCGGATTGGCCTTCTTGGCAGCAGCCAAAGGCTACAAGCTTATCATTACAATGCCTGCTTCAATGAGTCTTGAAAGAAGAACCATTCTTCTCTCTTTTGGAGCTGAGTTGGTTCTCACGGATCCTGCAAAGGGAATGAAAGGGGCAGTTCAGAAAGCTGAAGAGATATTAGCCAAGACTCCAAATGCCTATATCCTTCAGCAATTTGAAAACCCTGCCAATCCAAAGGTGCATTATGAAACCACTGGTCCAGAGATATGGAAAGGCACAGGAGGGAAAGTTGATGCATTTGTTAGTGGTATAGGGACTGGTGGTACCATAACAGGTGTTGGGAAATATCTTAAAGAGCAGAATTCCAATATCAAGCTGTATGGAGTGGAGCCAGTTGAAAGTCCCGTTCTGTCCGGAGGAAAGCCTGGTCCACACAAGATTCAAGGGATTGGTGCTGGTTTTGTTCCCGGGGTATTGGATGTTAGTCTTGTCGACGAAGTAATTCAAATATCAAGTGATGAAGCAATAGAAACTGCAAAGCTTCTTGCCCTTAAAGAAGGCCTATTTGTTGGAATATCATCTGGAGCTGCCGCGGCAGCTGCCATTAAGATAGCAAAACGACCAGAAAATGCTGGAAAGCTTATTGTTGTTGTTTTCCCTAGCTTTGGTGAGAGGTACCTATCCTCTGTGCTGTTTGAATCGGTGAGACGGGAGGCTGAAAGCCTGACTTTTGAACCATGA |
|  |  | Protein | MAVERSGIAKDITELIGKTPLVYLNKITDGSVARVAAKLELMEPCSSVKDRIGYSMIADAEEKGLITPGQSVLIEPTSGNTGIGLAFLAAAKGYKLIITMPASMSLERRTILLSFGAELVLTDPAKGMKGAVQKAEEILAKTPNAYILQQFENPANPKVHYETTGPEIWKGTGGKVDAFVSGIGTGGTITGVGKYLKEQNSNIKLYGVEPVESPVLSGGKPGPHKIQGIGAGFVPGVLDVSLVDEVIQISSDEAIETAKLLALKEGLFVGISSGAAAAAAIKIAKRPENAGKLIVVVFPSFGERYLSSVLFESVRREAESLTFEP* |
| *MsCSase15* | MS.gene26579.t1 | CDS | ATGGCTTCTACTTCCTTAATCAACTCATTGACTTTTACTTCCCGCACCCCCACTCTTCACTACTTAACCAGCCGCACCTCCACCTCATCACTCCCATTCGCTATCTCCCCCCGTTCCATCTCCACCAGAATCTCTTCCCCCGTTCTATGCAAGGCCGTCTCCGTCAATTCCCAAACCACCATTGAAGGTCTCAACATTGCCGAAGATGTTACTCAGCTCATAGGAAAAACACCAATGGTTTACCTGAATAGCATCGCAAAGGGTTCCGTTGCTAATATTGCTGCTAAGCTTGAGATTATGGAGCCTTGTTGTAGTGTCAAAGACAGGATAGGTTATAGTATGATACTTGATGCTGAGAAGAAAGGAGCCATAACACCTGGGAAGAGTGTTTTGGTGGAACCTACTAGTGGGAACACAGGAATTGGTCTTGCCTTTATAGCAGCTTCTAAAGGTTATAAACTCATTTTAACAATGCCTGCTTCCATGAGTTTGGAAAGACGAGTTCTTTTGAAAGCATTTGGAGCTGAGCTTGTTTTGACTGAGGCTGCGAAGGGTATGAATGGAGCAGTTCAAAAAGCTGAAGAAATTGTAAAAAGTACACCTGATGCATACATGCTTCAACAATTTGACAATCCTTCAAATCCCAAGATACATTTTGAGACAACTGGGCCAGAGATATGGGAAGATACAAGAGGCAAAATAGATATTTTAGTTGCAGGGATTGGAACTGGTGGAACCATTTCTGGAACCGGTCGATTCCTAAAACAACAAAATTCAAAAGTACAGGTTATTGGTGTAGAGCCTCTCGAAAGCAACATACTTTCAGGTGGAAAGCCAGGACCTCACAAAATTCAGGGTATTGGGGCTGGTTTTGTGCCCAGGAATTTGGATGAAGAAGTGCTTGATGAAGTTATAGCGATATCCAGTGATGAAGCTATTGAAACTACAAAGCAAATAGCACTTCAGGAAGGCTTGCTGGTGGGAATTTCTTCAGGTGCTGCTGCTGCAGCTGCATTGCAGGTTGCAAAGAGGCCTGAAAATGAGGGAAAGCTTATTGGGGTTGTATTCCCAAGCTTTGGTGAAAGATATTTGTCCACTGTTCTTTTCCAGAAAATTCGAGAAGAATGTGAGAACATGCAACCGGAGCCATGA |
|  |  | Protein | MASTSLINSLTFTSRTPTLHYLTSRTSTSSLPFAISPRSISTRISSPVLCKAVSVNSQTTIEGLNIAEDVTQLIGKTPMVYLNSIAKGSVANIAAKLEIMEPCCSVKDRIGYSMILDAEKKGAITPGKSVLVEPTSGNTGIGLAFIAASKGYKLILTMPASMSLERRVLLKAFGAELVLTEAAKGMNGAVQKAEEIVKSTPDAYMLQQFDNPSNPKIHFETTGPEIWEDTRGKIDILVAGIGTGGTISGTGRFLKQQNSKVQVIGVEPLESNILSGGKPGPHKIQGIGAGFVPRNLDEEVLDEVIAISSDEAIETTKQIALQEGLLVGISSGAAAAAALQVAKRPENEGKLIGVVFPSFGERYLSTVLFQKIREECENMQPEP* |
| *MsCSase16* | MS.gene26578.t1 | CDS | ATGGCTTCTTCTTCCTTAGTCAAGTCTTTTACTTGTGCTTTGCGTGCTCGCCGTACCAGCCACCGTTTCAGCTCAACCAGGATCTTTTCATCTTCCCCTGCCTTTGTCGAATCCCAAACCAGCAACGATGATCTCAATATTGCCAATGATATTACAGAGCTCATAGGAAAAACGCCAATGGTTTACCTGAAGAGTATCACTAAGGGCTCAGTTGCTAACATTGCTGCAAAGCTTGAGCTTATGGAGCCATGTTGCAGTGTCAAAGACAGGATAGGTTATAGCATGATTCTTGATGCCGAGCAGAAAGGACTCATAACACCTGGGAAGAGTATTTTGGTAGAACCTACCGGTGGGAACACAGGAATTGGTCTTGCCTTTATAGCAGCTACTAAAGGTTATAAACTCATTTTGACAATGCCTGTTTCAATGAGTTTAGAAAGAAGAGTTCTTATGAAAGCATTTGGAGCTGAGCTTGTTTTGACCGAATATTCCAAGGCTATGAGTGGAGCAGTTCAAAAAGCTGAAGAAATTGTAAAAAATACACCTAATGCATACATGCTTCAACAATTTGATAATCCTTCAAATCCTAAGGTGCATTATGAGAATACTGGGCCAGAGATATGGAAAGATACAAAAGGCAAAGTAGACATTTTAGTCTCGGCAATTGGAACCGGTGGAACTCTTTCTGGAGCTGGTAGATTCCTAAAACAACAAAATCCAAAAATAAAGTGCATCGGTGTAGAGCCTCTCGAAAGCAACATACTTTCAGGTGGAAAGCCAAGACCACACATAATTCAGGGTATTGGGGCTGGTTTTGTGCCCAAGAATTTGGATAAAGAAATCCTTGATGAAGTTATAGCGATATCCGGCGAAGAATCTGTCAAAACTGCAAGGCAAATAGCACTCCAGGAAGGCTTGCTGGTGGGAATTTCCTCAGGTTGTGCTGCTACAGCTGCATTGCAGGTTGCTAAGAGACCTGAAAATGAGGGAAAGCTTATTGTGGTTGTATTCCCAAGCTTCGGTGAAAGATATGTGTCTACTTCTCTTTTCCAGGAAGCTCGAGAAGAATGTGAGAAAATGCAACCGGAGCCATGA |
|  |  | Protein | MASSSLVKSFTCALRARRTSHRFSSTRIFSSSPAFVESQTSNDDLNIANDITELIGKTPMVYLKSITKGSVANIAAKLELMEPCCSVKDRIGYSMILDAEQKGLITPGKSILVEPTGGNTGIGLAFIAATKGYKLILTMPVSMSLERRVLMKAFGAELVLTEYSKAMSGAVQKAEEIVKNTPNAYMLQQFDNPSNPKVHYENTGPEIWKDTKGKVDILVSAIGTGGTLSGAGRFLKQQNPKIKCIGVEPLESNILSGGKPRPHIIQGIGAGFVPKNLDKEILDEVIAISGEESVKTARQIALQEGLLVGISSGCAATAALQVAKRPENEGKLIVVVFPSFGERYVSTSLFQEAREECEKMQPEP* |
| *MsCSase17* | MS.gene39577.t1 | CDS | ATGGCTTCTACTTCCTTAATCAACTCATTGACTTTTACTTCCCGCACCCCCACTCTTCACTACTTAACCAGCCGCACCTCCACCTCATCACTCCCATTCGCTATCTCCCCCCGTTCCATCTCCACCAGAATCTCTTCCCCCGTTCTATGCAAGGCCGTCTCCGTCAATTCCCAAACCACCATTGAAGGTCTCAACATTGCCGAAGATGTTACTCAGCTCATAGGAAAAACACCAATGGTTTACCTGAATAGCATCGCAAAGGGTTCCGTTGCTAATATTGCTGCTAAGCTTGAGATTATGGAGCCTTGTTGTAGTGTCAAAGACAGGATAGGTTATAGTATGATACTTGATGCTGAGAAGAAAGGAGCCATAACACCTGGGAAGAGTGTTTTGGTGGAACCTACTAGTGGGAACACAGGAATTGGTCTTGCCTTTATAGCAGCTTCTAAAGGTTATAAACTCATTTTAACAATGCCTGCTTCCATGAGTTTGGAAAGACGAGTTCTTTTGAAAGCATTTGGAGCTGAGCTTGTTTTGACTGAGGCTGCGAAGGGTATGAATGGAGCAGTTCAAAAAGCTGAAGAAATTGTAAAAAGTACACCTGATGCATACATGCTTCAACAATTTGACAATCCTTCAAATCCCAAGATACATTTTGAGACAACTGGGCCAGAGATATGGGAAGATACAAGAGGCAAAATAGATATTTTAGTTGCAGGGATTGGAACTGGTGGAACCATTTCTGGAACCGGTCGATTCCTAAAACAACAAAATTCAAAAGTACAGGTTATTGGTGTAGAGCCTCTCGAAAGCAACATACTTTCAGGTGGAAAGCCAGGACCTCACAAAATTCAGGGTATTGGGGCTGGTTTTGTGCCCAGGAATTTGGATGAAGAAGTGCTTGATGAAGTTATAGCGATATCCAGTGATGAAGCTATTGAAACTACAAAGCAAATAGCACTTCAGGAAGGCTTGCTGGTGGGAATTTCTTCAGGTGCTGCTGCTGCAGCTGCATTGCAGGTTGCAAAGAGGCCTGAAAATGAGGGAAAGCTTATTGGGGTTGTATTCCCAAGCTTTGGTGAAAGATATTTGTCCACTGTTCTTTTCCAGAAAATTCGAGAAGAATGTGAGAACATGCAACCGGAGCCATGA |
|  |  | Protein | MASTSLINSLTFTSRTPTLHYLTSRTSTSSLPFAISPRSISTRISSPVLCKAVSVNSQTTIEGLNIAEDVTQLIGKTPMVYLNSIAKGSVANIAAKLEIMEPCCSVKDRIGYSMILDAEKKGAITPGKSVLVEPTSGNTGIGLAFIAASKGYKLILTMPASMSLERRVLLKAFGAELVLTEAAKGMNGAVQKAEEIVKSTPDAYMLQQFDNPSNPKIHFETTGPEIWEDTRGKIDILVAGIGTGGTISGTGRFLKQQNSKVQVIGVEPLESNILSGGKPGPHKIQGIGAGFVPRNLDEEVLDEVIAISSDEAIETTKQIALQEGLLVGISSGAAAAAALQVAKRPENEGKLIGVVFPSFGERYLSTVLFQKIREECENMQPEP* |
| *MsCSase18* | MS.gene39578.t1 | CDS | ATGGCTTCTTCTTCCTTAGTCAAGTCTTTTACTTGTGCTTTGCGTGCTCGCCGTACCAGCCACCGTTTCAGCTCAACCAGGATCTTTTCATCTTCCCCTGCCTTTGTCGAATCCCAAACCAGCAACGATGATCTCAATATTGCCAATGATATTACAGAGCTCATAGGAAAAACGCCAATGGTTTACCTGAAGAGTATCACTAAGGGCTCAGTTGCTACTATTGCTGCTAAGCTTGAGCTTATGGAGCCATGTTGCAGTGTCAAAGACAGGATAGGTTATAGCATGATTCTTGATGCCGAGCAGAAAGGACTCATAACACCTGGGAAGAGTATTTTGGTAGAACCTACCGGTGGGAACACAGGAATTGGTCTTGCCTTTATAGCAGCTACTAAAGGTTATAAACTCATTTTGACAATGCCTGTTTCAATGAGTTTAGAAAGAAGAGTTCTTATGAAAGCATTTGGAGCTGAGCTTGTTTTGACCGAATATTCCAAGGCTATGAGTGGAGCAGTTCAAAAAGCTGAAGAAATTGTAAAAAATACACCTAATGCATACATGCTGCAACAATTTGATAATCCTTCAAATCCTAAGGTGCATTATGAGAATACTGGGCCAGAGATATGGAAAGATACAAAAGGCAAAGTAGACATTTTAGTCTCGGCAATTGGAACCGGTGGAACTCTTTCTGGAGCTGGTAGATTCCTAAAACAACAAAATCCAAAAATAAAGTGCATCGGTGTAGAGCCTCTCGAAAGCAACATACTTTCAGGTGGAAAGCCAAGACCACACATAATTCAGGGTATTGGGGCTGGTTTTGTGCCCAAGAATTTGGATAAAGAAATCCTTGATGAAGTTATAGCGATATCCGGCGAAGAATCTGTCAAAACTGCAAGGCAAATAGCACTCCAGGAAGGCTTGCTGGTGGGAATTTCCTCAGGTTGTGCTGCTACAGCTGCATTGCAGGTTGCTAAGAGACCTGAAAATGAGGGAAAGCTTATTGTGGTTGTATTCCCAAGCTTCGGTGAAAGATATGTGTCTACTTCTCTTTTCCAGGAAGCTCGAGAAGAATGTGAGAAAATGCAACCGGAGCCATGA |
|  |  | Protein | MASSSLVKSFTCALRARRTSHRFSSTRIFSSSPAFVESQTSNDDLNIANDITELIGKTPMVYLKSITKGSVATIAAKLELMEPCCSVKDRIGYSMILDAEQKGLITPGKSILVEPTGGNTGIGLAFIAATKGYKLILTMPVSMSLERRVLMKAFGAELVLTEYSKAMSGAVQKAEEIVKNTPNAYMLQQFDNPSNPKVHYENTGPEIWKDTKGKVDILVSAIGTGGTLSGAGRFLKQQNPKIKCIGVEPLESNILSGGKPRPHIIQGIGAGFVPKNLDKEILDEVIAISGEESVKTARQIALQEGLLVGISSGCAATAALQVAKRPENEGKLIVVVFPSFGERYVSTSLFQEAREECEKMQPEP* |
| *MsCSase19* | MS.gene47527.t1 | CDS | ATGGCTTCTACTTCCTTAATCAACTCATTGACTTTTACTTCCCGCACCCCCACTCTTCACTACTTAACCAGCCGCACCTCCACCTCATCACTCCCATTCGCTATCTCCCCCCGTTCCATCTCCACCAGAATCTCTTCCCCCGTTCTATGCAAGGCCGTCTCCGTCAATTCCCAAACCACCATTGAAGGTCTCAACATTGCCGAAGATGTTACTCAGCTCATAGGAAAAACACCAATGGTTTACCTGAATAGCATCGCAAAGGGTTCCGTTGCTAATATTGCTGCTAAGCTTGAGATTATGGAGCCTTGTTGTAGTGTCAAAGACAGGATAGGTTATAGTATGATACTTGATGCTGAGAAGAAAGGAGCCATAACACCTGGGAAGAGTGTTTTGGTGGAACCTACTAGTGGGAACACAGGAATTGGTCTTGCCTTTATAGCAGCTTCTAAAGGTTATAAACTCATTTTAACAATGCCTGCTTCCATGAGTTTGGAAAGACGAGTTCTTTTGAAAGCATTTGGAGCTGAGCTTGTTTTGACTGAGGCTGCGAAGGGTATGAATGGAGCAGTTCAAAAAGCTGAAGAAATTGTAAAAAGTACACCTGATGCATACATGCTTCAACAATTTGACAATCCTTCAAATCCCAAGATACATTTTGAGACAACTGGGCCAGAGATATGGGAAGATACAAGAGGCAAAATAGATATTTTAGTTGCAGGGATTGGAACTGGTGGAACCATTTCTGGAACCGGTCGATTCCTAAAACAACAAAATTCAAAAGTACAGGTTATTGGTGTAGAGCCTCTCGAAAGCAACATACTTTCAGGTGGAAAGCCAGGACCTCACAAAATTCAGGGTATTGGGGCTGGTTTTGTGCCCAGGAATTTGGATGAAGAAGTGCTTGATGAAGTTATAGCGATATCCAGTGATGAAGCTATTGAAACTACAAAGCAAATAGCACTTCAGGAAGGCTTGCTGGTGGGAATTTCTTCAGGTGCTGCTGCTGCAGCTGCATTGCAGGTTGCAAAGAGGCCTGAAAATGAGGGAAAGCTTATTGGGGTTGTATTCCCAAGCTTTGGTGAAAGATATTTGTCCACTGTTCTTTTCCAGAAAATTCGAGAAGAATGTGAGAACATGCAACCGGAGCCATGA |
|  |  | Protein | MASTSLINSLTFTSRTPTLHYLTSRTSTSSLPFAISPRSISTRISSPVLCKAVSVNSQTTIEGLNIAEDVTQLIGKTPMVYLNSIAKGSVANIAAKLEIMEPCCSVKDRIGYSMILDAEKKGAITPGKSVLVEPTSGNTGIGLAFIAASKGYKLILTMPASMSLERRVLLKAFGAELVLTEAAKGMNGAVQKAEEIVKSTPDAYMLQQFDNPSNPKIHFETTGPEIWEDTRGKIDILVAGIGTGGTISGTGRFLKQQNSKVQVIGVEPLESNILSGGKPGPHKIQGIGAGFVPRNLDEEVLDEVIAISSDEAIETTKQIALQEGLLVGISSGAAAAAALQVAKRPENEGKLIGVVFPSFGERYLSTVLFQKIREECENMQPEP* |
| *MsCSase20* | MS.gene47526.t1 | CDS | ATGGCTTCTTCTTCCTTAGTCAAGTCTTTGAATTATGCTTTGCGTGCTCGTGCTAGCCCTATCGGCCACTGTTTCAGCTTCACCAGGATCTTTTCATCTTCCCCTGTCTTTGTCGAATCCCAAACCAGCAACGATGATCTCAATATTGCCAATGATATTACAGAGCTCATAGGAAAAACGCCAATGGTTTACCTGAAGAGTATCACTAAGGGCTCAGTTGCTAACATTGCTGCAAAGCTTGAGCTTATGGAGCCATGTTGCAGTGTCAAAGACAGGATAGGTTATAGCATGATTCTTGATGCCGAGCAGAAAGGACTCATAACACCTGGGAAGAGTATTTTGGTAGAACCTACCGGTGGGAACACAGGAATTGGTCTTGCCTTTATAGCAGCTACTAAAGGTTATAAACTCATTTTGACAATGCCTGTTTCAATGAGTTTAGAAAGAAGAGTTCTTATGAAAGCATTTGGAGCTGAGCTTGTTTTGACCGAATATTCCAAGGCTATGAGTGGAGCAGTTCAAAAAGCTGAAGAAATTGTAAAAAATACACCTAATGCATACATGCTTCAACAATTTGATAATCCTTCAAATCCTAAGGTGCATTATGAGAATACTGGGCCAGAGATATGGAAAGATACAAAAGGCAAAGTAGACATTTTAGTCTCGGCAATTGGAACCGGTGGAACTCTTTCTGGAGCTGGTAGATTCCTAAAACAACAAAATCCAAAAATAAAGTGCATCGGTGTAGAGCCTCTCGAAAGCAACATACTTTCAGGTGGAAAGCCAAGACCACACATAATTCAGGGTATTGGGGCTGGTTTTGTGCCCAAGAATTTGGATAAAGAAATCCTTGATGAAGTTATAGCGATATCCGGCGAAGAATCTGTCAAAACTGCAAGGCAAATAGCACTCCAGGAAGGCTTGCTGGTGGGAATTTCCTCAGGTTGTGCTGCTACAGCTGCATTGCAGGTTGCTAAGAGACCTGAAAATGAGGGAAAGCTTATTGTGGTTGTATTCCCAAGCTTCGGTGAAAGATATGTGTCTACTTCTCTTTTCCAGGAAGCTCGAGAAGAATGTGAGAAAATGCAACCGGAGCCATGA |
|  |  | Protein | MASSSLVKSLNYALRARASPIGHCFSFTRIFSSSPVFVESQTSNDDLNIANDITELIGKTPMVYLKSITKGSVANIAAKLELMEPCCSVKDRIGYSMILDAEQKGLITPGKSILVEPTGGNTGIGLAFIAATKGYKLILTMPVSMSLERRVLMKAFGAELVLTEYSKAMSGAVQKAEEIVKNTPNAYMLQQFDNPSNPKVHYENTGPEIWKDTKGKVDILVSAIGTGGTLSGAGRFLKQQNPKIKCIGVEPLESNILSGGKPRPHIIQGIGAGFVPKNLDKEILDEVIAISGEESVKTARQIALQEGLLVGISSGCAATAALQVAKRPENEGKLIVVVFPSFGERYVSTSLFQEAREECEKMQPEP* |
| *MsCSase21* | MS.gene008717.t1 | CDS | ATGGCTTCTACTTCCTTAATCAACTCATTGACTTTTACTTCCCGCACCCCCACTCTTCACTACTTAACCAGCCGCACCTCCACCTCATCACTCCCATTCGCTATCTCCCCCCGTTCCATCTCCACCAGAATCTCTTCCCCCGTTCTATGCAAGGCCGTCTCCGTCAATTCCCAAACCACCATTGAAGGTCTCAACATTGCCGAAGATGTTACTCAGCTCATAGGAAAAACACCAATGGTTTACCTGAATAGCATCGCAAAGGGTTCCGTTGCTAATATTGCTGCTAAGCTTGAGATTATGGAGCCTTGTTGTAGTGTCAAAGACAGGATAGGTTATAGTATGATACTTGATGCTGAGAAGAAAGGAGCCATAACACCTGGGAAGAGTGTTTTGGTGGAACCTACTAGTGGGAATACAGGAATTGGTCTTGCCTTTATAGCAGCTTCTAAAGGTTATAAACTCATTTTAACAATGCCTGCTTCCATGAGTTTGGAAAGACGAGTTCTTTTGAAAGCATTTGGAGCTGAGCTTGTTTTGACTGAGGCTGCGAAGGGTATGAATGGAGCAGTTCAAAAAGCTGAAGAAATTGTAAAAAGTACACCTGATGCATACATGCTTCAACAATTTGACAATCCTTCAAATCCCAAGATACATTTTGAGACAACTGGGCCAGAGATATGGGAAGATACAAGAGGCAAAATAGATATTTTAGTTGCAGGGATTGGAACTGGTGGAACCATTTCTGGAACCGGTCGATTCCTAAAACAACAAAATTCAAAAGTACAGGTTATTGGTGTAGAGCCTCTCGAAAGCAACATACTTTCAGGTGGAAAGCCAGGACCTCACAAAATTCAGGGTATTGGGGCTGGTTTTGTGCCCAGGAATTTGGATGAAGAAGTGCTTGATGAAGTTATAGCGATATCCAGTGATGAAGCTATTGAAACTACAAAGCAAATAGCACTTCAGGAAGGCTTGCTGGTGGGAATTTCTTCAGGTGCTGCTGCTGCAGCTGCATTGCAGGTTGCAAAGAGGCCTGAAAATGAGGGAAAGCTTATTGGGGTTGTATTCCCAAGCTTTGGTGAAAGATATTTGTCCACTGTTCTTTTCCAGAAAATTCGAGAAGAATGTGAGAACATGCAACCGGAGCCATGA |
|  |  | Protein | MASTSLINSLTFTSRTPTLHYLTSRTSTSSLPFAISPRSISTRISSPVLCKAVSVNSQTTIEGLNIAEDVTQLIGKTPMVYLNSIAKGSVANIAAKLEIMEPCCSVKDRIGYSMILDAEKKGAITPGKSVLVEPTSGNTGIGLAFIAASKGYKLILTMPASMSLERRVLLKAFGAELVLTEAAKGMNGAVQKAEEIVKSTPDAYMLQQFDNPSNPKIHFETTGPEIWEDTRGKIDILVAGIGTGGTISGTGRFLKQQNSKVQVIGVEPLESNILSGGKPGPHKIQGIGAGFVPRNLDEEVLDEVIAISSDEAIETTKQIALQEGLLVGISSGAAAAAALQVAKRPENEGKLIGVVFPSFGERYLSTVLFQKIREECENMQPEP* |
| *MsCSase22* | MS.gene02466.t1 | CDS | ATGGCTTCTACTTCCTTAATCAACTCATTGACTTTTACTTCCCGCACCCCCACTCTTCACTACTTAACCAGCCGCACCTCCACCTCATCACTCCCATTCGCTATCTCCCCCCGTTCCATCTCCACCAGAATCTCTTCCCCCGTTCTATGCAAGGCCGTCTCCGTCAATTCCCAAACCACCATTGAAGGTCTCAACATTGCCGAAGATGTTACTCAGCTCATAGGAAAAACACCAATGGTTTACCTGAATAGCATCGCAAAGGGTTCCGTTGCTAATATTGCTGCTAAGCTTGAGATTATGGAGCCTTGTTGTAGTGTCAAAGACAGGATAGGTTATAGTATGATACTTGATGCTGAGAAGAAAGGAGCCATAACACCTGGGAAGAGTGTTTTGGTGGAACCTACTAGTGGGAATACAGGAATTGGTCTTGCCTTTATAGCAGCTTCTAAAGGTTATAAACTCATTTTAACAATGCCTGCTTCCATGAGTTTGGAAAGACGAGTTCTTTTGAAAGCATTTGGAGCTGAGCTTGTTTTGACTGAGGCTGCGAAGGGTATGAATGGAGCAGTTCAAAAAGCTGAAGAAATTGTAAAAAGTACACCTGATGCATACATGCTTCAACAATTTGACAATCCTTCAAATCCCAAGATACATTTTGAGACAACTGGGCCAGAGATATGGGAAGATACAAGAGGCAAAATAGATATTTTAGTTGCAGGGATTGGAACTGGTGGAACCATTTCTGGAACCGGTCGATTCCTAAAACAACAAAATTCAAAAGTACAGGTTATTGGTGTAGAGCCTCTCGAAAGCAACATACTTTCAGGTGGAAAGCCAGGACCTCACAAAATTCAGGGTATTGGGGCTGGTTTTGTGCCCAGGAATTTGGATGAAGAAGTGCTTGATGAAGTTATAGCGATATCCAGTGATGAAGCTATTGAAACTACAAAGCAAATAGCACTTCAGGAAGGCTTGCTGGTGGGAATTTCTTCAGGTGCTGCTGCTGCAGCTGCATTGCAGGTTGCAAAGAGGCCTGAAAATGAGGGAAAGCTTATTGGGGTTGTATTCCCAAGCTTTGGTGAAAGATATTTGTCCACTGTTCTTTTCCAGAAAATTCGAGAAGAATGTGAGAACATGCAACCGGAGCCATGA |
|  |  | Protein | MASTSLINSLTFTSRTPTLHYLTSRTSTSSLPFAISPRSISTRISSPVLCKAVSVNSQTTIEGLNIAEDVTQLIGKTPMVYLNSIAKGSVANIAAKLEIMEPCCSVKDRIGYSMILDAEKKGAITPGKSVLVEPTSGNTGIGLAFIAASKGYKLILTMPASMSLERRVLLKAFGAELVLTEAAKGMNGAVQKAEEIVKSTPDAYMLQQFDNPSNPKIHFETTGPEIWEDTRGKIDILVAGIGTGGTISGTGRFLKQQNSKVQVIGVEPLESNILSGGKPGPHKIQGIGAGFVPRNLDEEVLDEVIAISSDEAIETTKQIALQEGLLVGISSGAAAAAALQVAKRPENEGKLIGVVFPSFGERYLSTVLFQKIREECENMQPEP* |
| *MsCSase23* | MS.gene02467.t1 | CDS | ATGGCTTCTTCTTCCTTAGTCAAGTCTTTTACTTGTGCTTTGCGTGCTCGCCGTACCAGCCACCGTTTCAGCTCAACCAGGATCTTTTCATCTTCCCCTGCCTTTGTCGAATCCCAAACCAGCAACGATGATCTCAATATTGCCAATGATATTACAGAGCTCATAGGAAAAACGCCAATGGTTTACCTGAAGAGTATCACTAAGGGCTCAGTTGCTACTATTGCTGCTAAGCTTGAGCTTATGGAGCCATGTTGCAGTGTCAAAGACAGGATAGGTTATAGCATGATTCTTGATGCCGAGCAGAAAGGACTCATAACACCTGGGAAGAGTATTTTGGTAGAACCTACCGGTGGGAACACAGGAATTGGTCTTGCCTTTATAGCAGCTACTAAAGGTTATAAACTCATTTTGACAATGCCTGTTTCAATGAGTTTAGAAAGAAGAGTTCTTATGAAAGCATTTGGAGCTGAGCTTGTTTTGACCGAATATTCCAAGGCTATGAGTGGAGCAGTTCAAAAAGCTGAAGAAATTGTAAAAAATACACCTAATGCATACATGCTTCAACAATTTGATAATCCTTCAAATCCTAAGGTGCATTATGAGAATACTGGGCCAGAGATATGGAAAGATACAAAAGGCAAAGTAGACATTTTAGTCTCGGCAATTGGAACCGGTGGAACTCTTTCTGGAGCTGGTAGATTCCTAAAACAACAAAATCCAAAAATAAAGTGCATCGGTGTAGAGCCTCTCGAAAGCAACATACTTTCAGGTGGAAAGCCAAGACCACACATAATTCAGGGTATTGGGGCTGGTTTTGTGCCCAAGAATTTGGATAAAGAAATCCTTGATGAAGTTATAGCGATATCCGGCGAAGAATCTGTCAAAACTGCAAGGCAAATAGCACTCCAGGAAGGCTTGCTGGTGGGAATTTCCTCAGGTTGTGCTGCTACAGCTGCATTGCAGGTTGCTAAGAGACCTGAAAATGAGGGAAAGCTTATTGTGGTTGTATTCCCAAGCTTCGGTGAAAGATATGTGTCTACTTCTCTTTTCCAGGAAGCTCGAGAAGAATGTGAGAAAATGCAACCGGAGCCATGA |
|  |  | Protein | MASSSLVKSFTCALRARRTSHRFSSTRIFSSSPAFVESQTSNDDLNIANDITELIGKTPMVYLKSITKGSVATIAAKLELMEPCCSVKDRIGYSMILDAEQKGLITPGKSILVEPTGGNTGIGLAFIAATKGYKLILTMPVSMSLERRVLMKAFGAELVLTEYSKAMSGAVQKAEEIVKNTPNAYMLQQFDNPSNPKVHYENTGPEIWKDTKGKVDILVSAIGTGGTLSGAGRFLKQQNPKIKCIGVEPLESNILSGGKPRPHIIQGIGAGFVPKNLDKEILDEVIAISGEESVKTARQIALQEGLLVGISSGCAATAALQVAKRPENEGKLIVVVFPSFGERYVSTSLFQEAREECEKMQPEP* |
| *MsCSase24* | MS.gene92293.t1 | CDS | ATGGCGGTCGAAAGGTCTGGAATTGCTAAAGATGTTACAGAATTGATTGGCAAAACCCCATTAGTGTATCTAAATAGACTTGCAGATGGCTGTGTTGCCCGAGTTGCTGCAAAACTGGAATTGATGGAGCCATGCTCTAGTGTAAAAGACAGGATTGGCTACAGTATGATTGCTGATGCAGAAGAGAAGGGGCTTATCACACCTGGACAGAGTGTCCTCATTGAACCAACAAGTGGTAACACTGGCATTGGATTAGCCTTCATGGCAGCAGCCAAGGGATACAAGCTCATAATTACGATGCCTGCTTCAATGAGTCTTGAAAGAAGAATCATTCTATTAGCTTTTGGAGCTGAATTGGTTTTGACAGACCCTGCAAAGGGAATGAAAGGAGCTGTTCAAAAAGCTGAAGAGCTGTTGGCTAAGACACCCAATGCCTACATACTTCAACAATTTGAAAACCCTGCCAATCCTAAGGTACATTATGAAACCACCGGTCCAGAGATATGGAAAGGCACGGATGGGAAAATTGACGCATTTGTTTCTGGGATAGGTACTGGTGGTACCATAACCGGTGCTGGGAAATATCTTAAAGAGCAAAATTCCAATATAAAGCTGATTGGTGTGGAACCAGTGGAGAGTCCAGTGCTCTCAGGGGGAAAACCTGGTCCTCACAAGATTCAAGGGATTGGTGCTGGCTTTGTCCCTGGTGTCTTAGAAGTTAATCTTATTGATGAAGTCGTTCAAATATCAAGTGATGAAGCAATAGAAACCGCAAAGCTTCTTGCTCTTAAAGAAGGCCTATTTGTGGGAATATCTTCTGGAGCTGCAGCTGCCGCTGCAATTAAGATAGCCAAAAGGCCAGAAAATTCTGGGAAGCTTATTGTTGTCGTTTTTCCAAGCTTTGGCGAGAGGTACCTATCCTCTGTGCTATTTGAGTCAGTGAGACGGGAGGCTGAAACCATGACTTTCGAGCCCTGA |
|  |  | Protein | MAVERSGIAKDVTELIGKTPLVYLNRLADGCVARVAAKLELMEPCSSVKDRIGYSMIADAEEKGLITPGQSVLIEPTSGNTGIGLAFMAAAKGYKLIITMPASMSLERRIILLAFGAELVLTDPAKGMKGAVQKAEELLAKTPNAYILQQFENPANPKVHYETTGPEIWKGTDGKIDAFVSGIGTGGTITGAGKYLKEQNSNIKLIGVEPVESPVLSGGKPGPHKIQGIGAGFVPGVLEVNLIDEVVQISSDEAIETAKLLALKEGLFVGISSGAAAAAAIKIAKRPENSGKLIVVVFPSFGERYLSSVLFESVRREAETMTFEP* |
| *MsCSase25* | MS.gene37942.t1 | CDS | ATGGCGCCTGTGGCAGCGAGAAGTGCAGGCGCTGTTGGCGTCGGAACCGCCATCTTCATCTCCCTCGTCGCCGCATACTTCTTCTGCGACCGTTGCTGCAACCCATCGAAGAAGAAGAAGAAATCAAAGAATGGAATCATTGACGCCATTGGTAACACTCCTTTGATTCGAATCAACAGTCTCTCTGATGCCACTGGTTGCGAAATTCTTGGCAAGTGTGAGTTTTTGAATCCTGGAGGGAGTGTTAAAGATCGTGTTGCAGTTCAAATTATTCAAGAGGCTTTGGAATCTGGGCAGCTACGACGAGGTGGAATAGTTACTGAAGGGAGTGCTGGAAGCACTGCAATTAGCATTGCTACAGTTGCTCCTGCTTATGGATGCAAATGTCATGTGGTTATTCCAGATGATGCTGCCATTGAGAAGTCTCAAATTATTGAAGCACTTGGAGCAACCGTTGAAAGAGTACGACCAGTGTCAATCACACACAAAGACCATTTTGTCAATATTGCAAGAAGACGGGCATCTGAAGCAAATGAGTTTGCATTTAAGCATAGAAAATCACAACCGAATGGCACGGACTCGCAGCAAATAAATGGTTGTAAATCTGACGGACACGAGCACAGCACACTCTTTCCTAATGATTGTCAAGGCGGTTTCTTTGCTGATCAGTTTGAGAACCTTGCAAACTTCAGGGCCCATTATGAGGGTACAGGACCTGAGATTTGGGAACAGACAAATGGAAATTTAGATGCATTTGTTGCAGCAGCAGGCACTGGTGGTACTGTGGCTGGTGTTTCCAAGTTTCTTCAGGAAAAGAATCCAAACATCAAGTGTTACCTCATAGATCCTCCGGGTTCTGGCTTGTTTAATAAGGTAACGAGGGGGGTAATGTACACCAAAGAGGAGGCAGAAGGACGAAGACTTAAGAATCCATTTGACACTATAACAGAAGGAATCGGAATTAACAGGGTAACGAAGAATTTTGCAGAGGCAAAACTTGATGGGGCCTTTAGAGGAACAGATATTGAGGCTGTTGAAATGGCCAGGTTTCTCGTGAAGAATGATGGGCTTTTCCTTGGGAGTTCTTCTGCGATGAACTGTGTTGGAGCAGTTAGAATAGCGCAATCAATTGGCCCTGGTCACACAATTGTAACCATTCTTTGTGATAGTGGAATGAGACATTTGAGCAAGTTTTATAATGCTGAATACTTGTCTCAGCTTGGTTTGACACCTAAAGCAACTGGATTAGAGTTCTTGGGTATCAAATGA |
|  |  | Protein | MAPVAARSAGAVGVGTAIFISLVAAYFFCDRCCNPSKKKKKSKNGIIDAIGNTPLIRINSLSDATGCEILGKCEFLNPGGSVKDRVAVQIIQEALESGQLRRGGIVTEGSAGSTAISIATVAPAYGCKCHVVIPDDAAIEKSQIIEALGATVERVRPVSITHKDHFVNIARRRASEANEFAFKHRKSQPNGTDSQQINGCKSDGHEHSTLFPNDCQGGFFADQFENLANFRAHYEGTGPEIWEQTNGNLDAFVAAAGTGGTVAGVSKFLQEKNPNIKCYLIDPPGSGLFNKVTRGVMYTKEEAEGRRLKNPFDTITEGIGINRVTKNFAEAKLDGAFRGTDIEAVEMARFLVKNDGLFLGSSSAMNCVGAVRIAQSIGPGHTIVTILCDSGMRHLSKFYNAEYLSQLGLTPKATGLEFLGIK* |
| *MsCSase26* | MS.gene21844.t1 | CDS | ATGGCGGTCGAAAGGTCTGGAATTGCTAAAGATGTTACAGAATTGATTGGCAAAACCCCATTAGTGTATCTAAATAGACTTGCAGATGGCTGTGTTGCCCGAGTTGCTGCAAAACTGGAATTGATGGAGCCATGCTCTAGTGTAAAAGACAGGATTGGCTACAGTATGATTGCTGATGCAGAAGAGAAGGGGCTTATCACACCTGGACAGAGTGTCCTCATTGAACCAACAAGTGGTAACACTGGCATTGGATTAGCCTTCATGGCAGCAGCCAAGGGATACAAGCTCATAATTACGATGCCTGCTTCAATGAGTCTTGAAAGAAGAATCATTCTATTAGCTTTTGGAGCTGAATTGGTTTTGACAGACCCTGCAAAGGGAATGAAAGGAGCTGTTCAAAAAGCTGAAGAGCTGTTGGCTAAGACACCCAATGCCTACATACTTCAACAATTTGAAAACCCTGCCAATCCTAAGGTACATTATGAAACCACCGGTCCAGAGATATGGAAAGGCACAGATGGGAAAATTGACGCATTTGTTTCTGGGATAGGTACTGGTGGTACAATAACCGGTGCTGGGAAATATCTTAAAGAGCAAAATTCCAATATAAAGCTGATTGGTGTGGAACCAGTGGAGAGTCCAGTGCTCTCAGGGGGAAAACCTGGTCCTCACAAGATTCAAGGGATTGGTGCTGGCTTTGTCCCTGGTGTCTTAGAAGTTAATCTTATTGATGAAGTCGTTCAAATATCAAGTGATGAAGCAATAGAAACCGCAAAGCTTCTTGCTCTTAAAGAAGGCCTATTTGTGGGAATATCTTCTGGAGCTGCAGCTGCCGCTGCAATTAAGATAGCCAAAAGGCCAGAAAATGCTGGGAAGCTTATTGTTGTCGTTTTTCCAAGCTTTGGCGAGAGGTACCTATCCTCTGTGCTATTTGAGTCAGTGAGACGGGAGGCTGAAACCATGACTTTCGAGCCCTGA |
|  |  | Protein | MAVERSGIAKDVTELIGKTPLVYLNRLADGCVARVAAKLELMEPCSSVKDRIGYSMIADAEEKGLITPGQSVLIEPTSGNTGIGLAFMAAAKGYKLIITMPASMSLERRIILLAFGAELVLTDPAKGMKGAVQKAEELLAKTPNAYILQQFENPANPKVHYETTGPEIWKGTDGKIDAFVSGIGTGGTITGAGKYLKEQNSNIKLIGVEPVESPVLSGGKPGPHKIQGIGAGFVPGVLEVNLIDEVVQISSDEAIETAKLLALKEGLFVGISSGAAAAAAIKIAKRPENAGKLIVVVFPSFGERYLSSVLFESVRREAETMTFEP* |
| *MsCSase27* | MS.gene81336.t1 | CDS | ATGGCGCCTGTGGCAGCGAGAAGTGCAGGCGCTGTTGGCGTCGGAACCGCCATCTTCATCTCCCTCGTCGCCGCATACTTCTTCTGCGACCGTTGCTGCAACCCATCGAAGAAGAAGAAGAAATCAAAGAATGGAATCATTGACGCCATTGGTAACACTCCTTTGATTCGAATCAACAGTCTCTCTGATGCCACTGGTTGCGAAATTCTTGGCAAGTGTGAGTTTTTGAATCCTGGAGGGAGTGTTAAAGATCGTGTTGCAGTTCAAATTATTCAAGAGGCTTTGGAATTTGGGCAGCTACGACGAGGTGGAATAGTTACTGAAGGGAGTGCTGGAAGCACTGCAATTAGCATTGCTACAGTTGCTCCTGCTTATGGATGCAAATGTCATGTGGTTATTCCAGATGATGCTGCCATTGAGAAGTCTCAAATTATTGAAGCACTTGGAGCAACCGTTGAAAGAGTACGACCAGTGTCAATCACACACAAAGACCATTTTGTCAATATTGCAAGAAAACGGGCATCTGAAGCAAATGAGTTTGCATTTAAGCATAGAAAATCACAACCGAATGGCACGGACTCGCAGCAAATAAATGGTTGTAAATCTGATGGACACGAGCACAGCACACTCTTTCCTAATGATTGTCAAGGCGGTTTCTTTGCTGATCAGTTTGAGAACCTTGCAAACTTCAGGGCCCATTATGAAGGTACAGGACCTGAGATTTGGGAACAGACAAATGGAAATTTAGATGCATTTATTGCAGCAGCAGGCACTGGTGGTACTGTGGCTGGTGTTTCCAAGTTTCTTCAGGAAAAGAATCCAAACATCAAGTGTTACCTCATAGATCCTCCGGGTTCTGGCTTGTTTAATAAGGTAACGAGGGGGGTAATGTACACCAAAGAGGAGGCAGAAGGACGAAGACTTAAGAATCCATTTGACACTATAACAGAAGGAATCGGAATTAACAGGGTAACGAAGAATTTTGCAGAGGCAAAACTTGATGGGGCCTTTAGAGGAACAGATATTGAGGCTGTTGAAATGGCCAGGTTTCTCGTGAAGAATGATGGGCTTTTCCTTGGGAGTTCTTCTGCGATGAACTGTGTTGGAGCAGTTAGAATAGCGCAATCAATTGGCCCTGGTCACACAATTGTAACCATTCTTTGTGATAGTGGAATGAGACATTTGAGCAAGTTTTATAATGCTGAATACTTGTCTCAGCTTGGTTTGACACCTAAAGCAACTGGATTAGAGTTCTTGGGTATCAAATGA |
|  |  | Protein | MAPVAARSAGAVGVGTAIFISLVAAYFFCDRCCNPSKKKKKSKNGIIDAIGNTPLIRINSLSDATGCEILGKCEFLNPGGSVKDRVAVQIIQEALEFGQLRRGGIVTEGSAGSTAISIATVAPAYGCKCHVVIPDDAAIEKSQIIEALGATVERVRPVSITHKDHFVNIARKRASEANEFAFKHRKSQPNGTDSQQINGCKSDGHEHSTLFPNDCQGGFFADQFENLANFRAHYEGTGPEIWEQTNGNLDAFIAAAGTGGTVAGVSKFLQEKNPNIKCYLIDPPGSGLFNKVTRGVMYTKEEAEGRRLKNPFDTITEGIGINRVTKNFAEAKLDGAFRGTDIEAVEMARFLVKNDGLFLGSSSAMNCVGAVRIAQSIGPGHTIVTILCDSGMRHLSKFYNAEYLSQLGLTPKATGLEFLGIK* |
| *MsCSase28* | MS.gene020786.t1 | CDS | ATGGCGCCTGTGGCAGCGAGAAGTGCAGGCGCTGTTGGCGTCGGAACCGCCATCTTCATCTCCCTCGTCGCCGCATACTTCTTCTGCGACCGTTGCTGCAACCCATCGAAGAAGAAGAAGAAATCAAAGAATGGAATCATTGACGCCATTGGTAACACTCCTTTGATTCGAATCAACAGTCTCTCTGATGCCACTGGTTGCGAAATTCTTGGCAAGTGTGAGTTTTTGAATCCTGGAGGGAGTGTTAAAGATCGTGTTGCAGTTCAAATTATTCAAGAGGCTTTGGAATCTGGGCAGCTACGACGAGGTGGAATAGTTACTGAAGGGAGTGCTGGAAGCACTGCAATTAGCATTGCTACAGTTGCTCCTGCTTATGGATGCAAATGTCATGTGGTTATTCCAGATGATGCTGCCATTGAGAAGTCTCAAATTATTGAAGCACTTGGAGCAACCGTTGAAAGAGTACGACCAGTGTCAATCACACACAAAGACCATTTTGTCAATATTGCAAGAAGACGGGCATCTGAAGCAAATGAGTTTGCATTTAAGCATAGAAAATCACAACCGAATGGCACGGACTCGCAGCAAATAAATGGTTGTAAATCTGACGGACACGAGCACAGCACACTCTTTCCTAATGATTGTCAAGGCGGTTTCTTTGCTGATCAGTTTGAGAACCTTGCAAACTTCAGGGCCCATTATGAGGGTACAGGACCTGAGATTTGGGAACAGACAAATGGAAATTTAGATGCATTTGTTGCAGCAGCAGGCACTGGTGGTACTGTGGCTGGTGTTTCCAAGTTTCTTCAGGAAAAGAATCCAAACATCAAGTGTTACCTCATAGATCCTCCGGGTTCTGGCTTGTTTAATAAGGTAACGAGGGGGGTAATGTACACCAAAGAGGAGGCAGAAGGACGAAGACTTAAGAATCCATTTGACACTATAACAGAAGGAATCGGAATTAACAGGGTAACGAAGAATTTTGCAGAGGCAAAACTTGATGGGGCCTTTAGAGGAACAGATATTGAGGCTGTTGAAATGGCCAGGTTTCTCGTGAAGAATGATGGGCTTTTCCTTGGGAGTTCTTCTGCGATGAACTGTGTTGGAGCAGTTAGAATAGCGCAATCAATTGGCCCTGGTCACACAATTGTAACCATTCTTTGTGATAGTGGAATGAGACATTTGAGCAAGTTTTATAATGCTGAATACTTGTCTCAGCTTGGTTTGACACCTAAAGCAACTGGATTAGAGTTCTTGGGTATCAAATGA |
|  |  | Protein | MAPVAARSAGAVGVGTAIFISLVAAYFFCDRCCNPSKKKKKSKNGIIDAIGNTPLIRINSLSDATGCEILGKCEFLNPGGSVKDRVAVQIIQEALESGQLRRGGIVTEGSAGSTAISIATVAPAYGCKCHVVIPDDAAIEKSQIIEALGATVERVRPVSITHKDHFVNIARRRASEANEFAFKHRKSQPNGTDSQQINGCKSDGHEHSTLFPNDCQGGFFADQFENLANFRAHYEGTGPEIWEQTNGNLDAFVAAAGTGGTVAGVSKFLQEKNPNIKCYLIDPPGSGLFNKVTRGVMYTKEEAEGRRLKNPFDTITEGIGINRVTKNFAEAKLDGAFRGTDIEAVEMARFLVKNDGLFLGSSSAMNCVGAVRIAQSIGPGHTIVTILCDSGMRHLSKFYNAEYLSQLGLTPKATGLEFLGIK* |
| *MsCSase29* | MS.gene000153.t1 | CDS | ATGGCGGTCGAAAGGTCTGGAATTGCTAAAGATGTTACAGAATTGATTGGCAAAACCCCATTAGTGTATCTAAATAGACTTGCAGATGGCTGTGTTGCCCGAGTTGCTGCAAAACTGGAATTGATGGAGCCATGCTCTAGTGTAAAAGACAGGATTGGCTACAGTATGATTGCTGATGCAGAAGAGAAGGGGCTTATCACACCTGGACAGAGTGTCCTCATTGAACCAACAAGTGGTAACACTGGCATTGGATTAGCCTTCATGGCAGCAGCCAAGGGATACAAGCTCATAATTACGATGCCTGCTTCAATGAGTCTTGAAAGAAGAATCATTCTATTAGCTTTTGGAGCTGAATTGGTTTTGACAGACCCTGCAAAGGGAATGAAAGGAGCTGTTCAAAAAGCTGAAGAGCTGTTGGCTAAGACACCCAATGCCTACATACTTCAACAATTTGAAAACCCTGCCAATCCTAAGGTACATTATGAAACCACCGGTCCAGAGATATGGAAAGGCACGGATGGGAAAATTGACGCATTTGTTTCTGGGATAGGTACTGGTGGTACCATAACCGGTGCTGGGAAATATCTTAAAGAGCAAAATTCCAATATAAAGCTGATTGGTGTGGAACCAGTGGAGAGTCCAGTGCTCTCAGGGGGAAAACCTGGTCCTCACAAGATTCAAGGGATTGGTGCTGGCTTTGTCCCTGGTGTCTTAGAAGTTAATCTTATTGATGAAGTCGTTCAAATATCAAGTGATGAAGCAATAGAAACCGCAAAGCTTCTTGCTCTTAAAGAAGGCCTATTTGTGGGAATATCTTCTGGAGCTGCAGCTGCCGCTGCAATTAAGATAGCCAAAAGGCCAGAAAATTCTGGGAAGCTTATTGTTGTCGTTTTTCCAAGCTTTGGCGAGAGGTACCTATCCTCTGTGCTATTTGAGTCAGTGAGACGGGAGGCTGAAACCATGACTTTCGAGCCCTGA |
|  |  | Protein | MAVERSGIAKDVTELIGKTPLVYLNRLADGCVARVAAKLELMEPCSSVKDRIGYSMIADAEEKGLITPGQSVLIEPTSGNTGIGLAFMAAAKGYKLIITMPASMSLERRIILLAFGAELVLTDPAKGMKGAVQKAEELLAKTPNAYILQQFENPANPKVHYETTGPEIWKGTDGKIDAFVSGIGTGGTITGAGKYLKEQNSNIKLIGVEPVESPVLSGGKPGPHKIQGIGAGFVPGVLEVNLIDEVVQISSDEAIETAKLLALKEGLFVGISSGAAAAAAIKIAKRPENSGKLIVVVFPSFGERYLSSVLFESVRREAETMTFEP* |
| *MsCSase30* | MS.gene024871.t1 | CDS | ATGGCGGTCGAAAGGTCTGGAATTGCTAAAGATGTTACAGAATTGATTGGCAAAACCCCATTAGTGTATCTAAATAGACTTGCAGATGGCTGTGTTGCCCGAGTTGCTGCAAAACTGGAATTGATGGAGCCATGCTCTAGTGTAAAAGACAGGATTGGCTACAGTATGATTGCTGATGCAGAAGAGAAGGGGCTTATCACACCTGGACAGAGTGTCCTCATTGAACCAACAAGTGGTAACACTGGCATTGGATTAGCCTTCATGGCAGCAGCCAAGGGATACAAGCTCATAATTACGATGCCTGCTTCAATGAGTCTTGAAAGAAGAATCATTCTATTAGCTTTTGGAGCTGAATTGGTTTTGACAGACCCTGCAAAGGGAATGAAAGGAGCTGTTCAAAAAGCTGAAGAGCTGTTGGCTAAGACACCCAATGCCTACATACTTCAACAATTTGAAAACCCTGCCAATCCTAAGGTACATTATGAAACCACCGGTCCAGAGATATGGAAAGGCACGGATGGGAAAATTGACGCATTTGTTTCTGGGATAGGTACTGGTGGTACCATAACCGGTGCTGGGAAATATCTTAAAGAGCAAAATTCCAATATAAAGCTGATTGGTGTGGAACCAGTGGAGAGTCCAGTGCTCTCAGGGGGAAAACCTGGTCCTCACAAGATTCAAGGGATTGGTGCTGGCTTTGTCCCTGGTGTCTTAGAAGTTAATCTTATTGATGAAGTCGTTCAAATATCAAGTGATGAAGCAATAGAAACCGCAAAGCTTCTTGCTCTTAAAGAAGGCCTATTTGTGGGAATATCTTCTGGAGCTGCAGCTGCCGCTGCAATTAAGATAGCCAAAAGGCCAGAAAATTCTGGGAAGCTTATTGTTGTCGTTTTTCCAAGCTTTGGCGAGAGGTACCTATCCTCTGTGCTATTTGAGTCAGTGAGACGGGAGGCTGAAACCATGACTTTCGAGCCCTGA |
|  |  | Protein | MAVERSGIAKDVTELIGKTPLVYLNRLADGCVARVAAKLELMEPCSSVKDRIGYSMIADAEEKGLITPGQSVLIEPTSGNTGIGLAFMAAAKGYKLIITMPASMSLERRIILLAFGAELVLTDPAKGMKGAVQKAEELLAKTPNAYILQQFENPANPKVHYETTGPEIWKGTDGKIDAFVSGIGTGGTITGAGKYLKEQNSNIKLIGVEPVESPVLSGGKPGPHKIQGIGAGFVPGVLEVNLIDEVVQISSDEAIETAKLLALKEGLFVGISSGAAAAAAIKIAKRPENSGKLIVVVFPSFGERYLSSVLFESVRREAETMTFEP* |
| *MsCSase31* | MS.gene013867.t1 | CDS | ATGGCAGAGAGAAATGCTTCTGCCATTAAGGTTGTTTCAAGAAAATCTTTATGGTTCTTTTTCACAACAACAACGTTATTTTTCATCATGTCATGGCTCTTTGTTCTCCGTTCCACACGTTCAGATTCAACACCAGCCACCATCTCAGAGCTTCTTTCCACCACCTCCAACGACGGTTCAGATTCCGTAACATCTCAATCACAAAACTCTGAATCCTCCTTCAGCAATAGAGCAATCCTCGTAAACACCGAAACAAAAACACCAGAAACTCCAATATGCAAAACTGAAACAACCATCATATCAAACGACGACACCAAGATCAATAAATCCAAAGTTGTTTTGAAAATTTTCATGTATGATTTACCTCCCGAGTTTCATTTCGGGTTATTAGATTGGAAAGGCGATGAAAAGACGAAAAACGTTTGGCCTGATATGAAAACCAAGATACCACATTACCCTGGCGGTTTGAATTTACAACACAGCATTGAATATTGGCTCACATTGGACATTCTTGCTTCAGAATTGCCAGAAATTTACCCTGCTAGAACTGTAATCAGAGTTAGAAACTCGACCGAAGCCGATGTTATATTTGTGCCATTTTTTTCATCTTTGACTTATAACAGACACTCCAAAACAGGTCCACGTGAAAAGAGGAGTAGAAACAAGGTGTTGCAAGAGAAGTTGGTGAGGTATTTGATGAGTCAAGAGGAATGGAAAAGGTCTGGTGGAAGGGATCATTTGATCTTGGCTCATCATCCTAATAGTGGAGTGAGGAATGCAACAAATGGCATGCGATCTTCGAAATTCTGCCTCAACATAGCCGGCGACACGCCATCATCAAACCGCTTATTCGATGCCATTGCAAGTCACTGTGTTCCTGTCATAATCAGTGATGAAATTGAGCTTCCATATGAGGATGTTCTTGACTACTCCAAGTTCTGTGTATTTGTTCGCACAAGGGATGCTGTTAAAAAGAAGTATCTGATAAACTTCATTAGGAGTATTGGCAAAGATGAGTGGACAAGAATGTGGAACAGATTGAAAGAAGTTGAAAAATTCTTTGAATTTCAGTTCCCTTCTAAGGAAGGTGATGCTGTTCAAATGATTTGGCAAGCTGTTTCACGTAAGTTGATTGGAAATACACCGATGATATATCTGAATAAAGTTACAGATGGATGTGTAGCAAATGTTGCTGCAAAACTTGAATCCATGGAACCTTGCAGAAGTGTCAAGGATAGAATTGGCTATAGCATGTTAGCTGATGCTGAAGAGAGTGGAGCAATTTCTCCGGGGAAGACTGTTTTAGTTGAACCTACAACTGGAAATACGGGACTTGGCCTTGCTTTTGTTGCCGCAACGAAGGGTTATAAACTGATAGTCACAATGCCTGCTTCTGTTAATATTGAGAGGAGAATCCTTTTACGAACATTTGGTGCAGAGGTTGTTTTGACGAATGCGGAGAAGGGGCTGAAAGGGGCTGTTGACAAAGCTGAAGAAATTGTCTATGCCACACCCAATGCTTACATGTTTCGACAGTTTGATAACAGGAATAACACAAAGATCCACTTCGAAACTACAGGGCCTGAGATATGGGAGGATACAATGGGTAATGTTGATTTATTGGTTGCTGCTATTGGAACTGGCGGCACTATTACAGGCACTGGACAATATCTGAAAATGATGAACAAAAAAATAAAGGTGGTCGGGGTGGAACCTGCAGATAGAAGTATAATCTCAGGAGACAATCCAGGTTTCATACCAAGCATTTTGGATATCAAACTGCTTGATGAAGTCGTCAAGGTTACCAATGTTGAAGCTATTGAAATGGCAAGGAGATTAGCATTGGAAGAAGGATTACTGGTTGGGATTTCTTCAGGAGCTGCAGCAGCAGCTGCGATAACTTTAGCAAGACGGCCTGAAAATTCTGGAAAACTTATAGCGATGAAGAGTGGTTTATGTACTCCCCTTCACAGAAGTGGCAAATGA |
|  |  | Protein | MAERNASAIKVVSRKSLWFFFTTTTLFFIMSWLFVLRSTRSDSTPATISELLSTTSNDGSDSVTSQSQNSESSFSNRAILVNTETKTPETPICKTETTIISNDDTKINKSKVVLKIFMYDLPPEFHFGLLDWKGDEKTKNVWPDMKTKIPHYPGGLNLQHSIEYWLTLDILASELPEIYPARTVIRVRNSTEADVIFVPFFSSLTYNRHSKTGPREKRSRNKVLQEKLVRYLMSQEEWKRSGGRDHLILAHHPNSGVRNATNGMRSSKFCLNIAGDTPSSNRLFDAIASHCVPVIISDEIELPYEDVLDYSKFCVFVRTRDAVKKKYLINFIRSIGKDEWTRMWNRLKEVEKFFEFQFPSKEGDAVQMIWQAVSRKLIGNTPMIYLNKVTDGCVANVAAKLESMEPCRSVKDRIGYSMLADAEESGAISPGKTVLVEPTTGNTGLGLAFVAATKGYKLIVTMPASVNIERRILLRTFGAEVVLTNAEKGLKGAVDKAEEIVYATPNAYMFRQFDNRNNTKIHFETTGPEIWEDTMGNVDLLVAAIGTGGTITGTGQYLKMMNKKIKVVGVEPADRSIISGDNPGFIPSILDIKLLDEVVKVTNVEAIEMARRLALEEGLLVGISSGAAAAAAITLARRPENSGKLIAMKSGLCTPLHRSGK* |
| *MsCSase32* | MS.gene023918.t1 | CDS | ATGGCTTCTCTAATGAGCTTCCTCAAGAGAACCTCTTCTTCATCATCATCATTAGCAGTTGCATGCCAGCATCCAATGATGATGAGGAGGTTGATGACAACAACAGCAACCAGTGTTGATTCTTCATCTTTTGCTCAGAGAATCAGAGATTTGCCTAAAGATCTTCCTGGTACCAACATCAAAAAACATGTTTCACAGCTTATTGGTAGGACTCCACTTGTTTATCTTAACAAAGTAACTGAAGGATGTGGTGCTTATATTGCTGTTAAGCAAGAGATGATGCAGCCTACTGCTAGCATCAAAGACAGACCGGCACTTGCTATGATGGAAGATGCTGAGAAAAAGAATTTGATAACTCCTGGAAAGACAATTCTGATAGAGCCAACATCAGGCAACATGGGAATCAGTTTGGCATTTATGGCTGCCATGAAGGGATATAAGATGGTTTTGACTATGCCATCTTACACAAGCTTGGAAAGAAGGGTTTGTATGAGAGCATTTGGAGCTGAATTGATTCTCACTGATCCAACCAAAGGAATGGGAGGAACAGTGAAAAAGGCTTATGACCTTTTGGAATCCACACCAAATGCTTTTATGCTGCAACAATTTTCCAACCCAGCCAATACTAAGGTGCATTTCGAAACAACAGGTCCTGAGATATGGGAGGATACAAATGGACAAGTTGACATATTTGTGATGGGAATAGGTAGCGGAGGCACGGTTTCCGGGGTTGGACAATATCTTAAATCACAAAACCCTAATGTTAAGATATATGGAGTGGAGCCATCTGAAAGTAATGTGCTGAATGGTGGTAAACCTGGTCCTCATCAAATAACTGGAAATGGTGTTGGATTCAAACCAGACATATTGGACATGGATGTAATGGAAAAAGTCCTAGAAGTTAGCAGTGAAGATGCGGTTAACATGGCTAGGACATTGGCCTTGAAAGAAGGACTCATGGTTGGTATATCGTCCGGAGCAAACACAGTTGCAGCACTCAGATTGGCTAATCTTCCTGAAAACAAAGGCAAACTTATAGTGACTGTTCATCCAAGTTTCGGGGAACGATACCTGTCATCTGTCTTGTTCCAAGACCTTCGGACAGAAGCTGAAAACATGCAGCCAGTTTCGGTCGATTAA |
|  |  | Protein | MASLMSFLKRTSSSSSSLAVACQHPMMMRRLMTTTATSVDSSSFAQRIRDLPKDLPGTNIKKHVSQLIGRTPLVYLNKVTEGCGAYIAVKQEMMQPTASIKDRPALAMMEDAEKKNLITPGKTILIEPTSGNMGISLAFMAAMKGYKMVLTMPSYTSLERRVCMRAFGAELILTDPTKGMGGTVKKAYDLLESTPNAFMLQQFSNPANTKVHFETTGPEIWEDTNGQVDIFVMGIGSGGTVSGVGQYLKSQNPNVKIYGVEPSESNVLNGGKPGPHQITGNGVGFKPDILDMDVMEKVLEVSSEDAVNMARTLALKEGLMVGISSGANTVAALRLANLPENKGKLIVTVHPSFGERYLSSVLFQDLRTEAENMQPVSVD* |
| *MsCSase33* | MS.gene054826.t1 | CDS | ATGGCTTCTCTAATGAGCTTCCTCAAGAGAACCTCTTCTTCATCATCATCATTAGCAGTTGCATGCCAGCATCCAATGATGATGAGGAGGTTGATGACAACAACAGCAACCAGTGTTGATTCTTCATCTTTTGCTCAGAGAATCAGAGATTTGCCTAAAGATCTTCCTGGTACCAACATCAAAAAACATGTTTCACAGCTTATTGGTAGGACTCCACTTGTTTATCTTAACAAAGTAACTGAAGGATGTGGTGCTTATATTGCTGTTAAGCAAGAGATGATGCAGCCTACTGCTAGCATCAAAGACAGACCGGCACTTGCTATGATGGAAGATGCTGAGAAAAAGAATTTGATAACTCCTGGAAAGACAATTCTGATAGAGCCAACATCAGGCAACATGGGAATCAGTTTGGCATTTATGGCTGCCATGAAGGGATATAAGATGGTTTTGACTATGCCATCTTACACAAGCTTGGAAAGAAGGGTTTGTATGAGAGCATTTGGAGCTGAATTGATTCTCACGGATCCAACGAAAGGAATGGGAGGAACAGTGAAAAAGGCTTATGACCTTTTGGAATCCACACCAAATGCTTTTATGCTGCAACAATTTTCCAACCCAGCCAATACTAAGGTGCATTTCGAAACAACAGGTCCTGAGATATGGGAGGATACAAATGGACAAGTTGACATATTTGTGATGGGAATAGGTAGCGGAGGCACGGTTTCCGGGGTTGGACAATATCTTAAATCACAAAACCCTAATGTTAAGATATATGGAGTGGAGCCATCGGAAAGTAATGTGCTGAATGGTGGTAAACCTGGTCCTCATCAAATAACTGGAAATGGTGTTGGATTCAAACCAGACATATTGGACATGGATGTAATGGAAAAAGTCCTAGAAGTTAGCAGTGAAGATGCAGTTAACATGGCTAGGACATTGGCCTTGAAAGAAGGACTCATGGTTGGTATATCGTCTGGAGCAAACACAGTTGCAGCACTCAGATTGGCTAACCTTCCTGAGAACAAAGGCAAACTGATTGTGGTAAGTACAATTTTCCTTAAAATATGCAAGCAAATGACACTTCTGTCTTTTTTATCACTGTTCATCCAAGTTTCGGGGAACGATATCTGTCATCCGTCTTGTTCCAAGACCTTCGAACAGAAGCTGAAAACATGCAGCCAGTCTCGGTCGATTAATGTCTTCCACGAGTTTTCATACAACTAG |
|  |  | Protein | MASLMSFLKRTSSSSSSLAVACQHPMMMRRLMTTTATSVDSSSFAQRIRDLPKDLPGTNIKKHVSQLIGRTPLVYLNKVTEGCGAYIAVKQEMMQPTASIKDRPALAMMEDAEKKNLITPGKTILIEPTSGNMGISLAFMAAMKGYKMVLTMPSYTSLERRVCMRAFGAELILTDPTKGMGGTVKKAYDLLESTPNAFMLQQFSNPANTKVHFETTGPEIWEDTNGQVDIFVMGIGSGGTVSGVGQYLKSQNPNVKIYGVEPSESNVLNGGKPGPHQITGNGVGFKPDILDMDVMEKVLEVSSEDAVNMARTLALKEGLMVGISSGANTVAALRLANLPENKGKLIVVSTIFLKICKQMTLLSFLSLFIQVSGNDICHPSCSKTFEQKLKTCSQSRSINVFHEFSYN* |
| *MsCSase34* | MS.gene058651.t1 | CDS | ATGGCTTCTCTAATGAGCTTCCTCAAGAGAACCTCTTCTTCATCATCATCATTAGCAGTTGCATGCCAGCATCCAATGATGATGAGGAGGTTGATGACAACAACAGCAACCAGTGTTGATTCTTCATCTTTTGCTCAGAGAATCAGAGATTTGCCTAAAGATCTTCCTGGTACCAACATCAAAAAACATGTTTCACAGCTTATTGGTAGGACTCCACTTGTTTATCTTAACAAAGTAACTGAAGGATGTGGTGCTTATATTGCTGTTAAGCAAGAGATGATGCAGCCTACTGCTAGCATCAAAGACAGACCGGCACTTGCTATGATGGAAGATGCTGAGAAAAAGAATTTGATAACTCCTGGAAAGACAATTCTGATAGAGCCAACATCAGGCAACATGGGAATCAGTTTGGCATTTATGGCTGCCATGAAGGGATATAAGATGGTTTTGACTATGCCATCTTACACAAGCTTGGAAAGAAGGGTTTGTATGAGAGCATTTGGAGCTGAATTGATTCTCACTGATCCAACCAAAGGAATGGGAGGAACAGTGAAAAAGGCTTATGACCTTTTGGAATCCACACCAAATGCTTTTATGCTGCAACAATTTTCCAACCCAGCCAATACTAAGGTGCATTTCGAAACAACAGGTCCTGAGATATGGGAGGATACAAATGGACAAGTTGACATATTTGTGATGGGAATAGGTAGCGGAGGCACGGTTTCCGGGGTTGGACAATATCTTAAATCACAAAACCCTAATGTTAAGATATATGGAGTGGAGCCATCTGAAAGTAATGTGCTGAATGGTGGTAAACCTGGTCCTCATCAAATAACTGGAAATGGTGTTGGATTCAAACCAGACATACTGGACATGGATGTAATGGAAAAAGTCCTAGAAGTTAGCAGTGAAGATGCAGTTAACATGGCTAGGACATTGGCCTTGAAAGAAGGACTCATGGTTGGTATATCGTCTGGAGCAAACACAGTTGCAGCACTCAGATTGGCTAACCTACCTGAGAACAAAGGCAAACTGATTGTGACTGTTCATCCAAGTTTCGGGGAACGATACCTGTCATCCGTCTTGTTCCAAGACCTTCGGACAGAAGCTGAAAACATGCAGCCAGTCTCGGTCGATTAA |
|  |  | Protein | MASLMSFLKRTSSSSSSLAVACQHPMMMRRLMTTTATSVDSSSFAQRIRDLPKDLPGTNIKKHVSQLIGRTPLVYLNKVTEGCGAYIAVKQEMMQPTASIKDRPALAMMEDAEKKNLITPGKTILIEPTSGNMGISLAFMAAMKGYKMVLTMPSYTSLERRVCMRAFGAELILTDPTKGMGGTVKKAYDLLESTPNAFMLQQFSNPANTKVHFETTGPEIWEDTNGQVDIFVMGIGSGGTVSGVGQYLKSQNPNVKIYGVEPSESNVLNGGKPGPHQITGNGVGFKPDILDMDVMEKVLEVSSEDAVNMARTLALKEGLMVGISSGANTVAALRLANLPENKGKLIVTVHPSFGERYLSSVLFQDLRTEAENMQPVSVD* |
| *MsCSase35* | MS.gene52446.t1 | CDS | ATGGCTTCTCTAATGAGCTTCCTCAAGAGAACCTCTTCTTCATCATCATCATTAGCAGTTGCATGCCAGCATCCAATGATGATGAGGAGGTTGATGACAACAACAGCAACCAGTGTTGATTCTTCATCTTTTGCTCAGAGAATCAGAGATTTGCCTAAAGATCTTCCTGGTACCAACATCAAAAAACATGTTTCACAGCTTATTGGTAGGACTCCACTTGTTTATCTTAACAAAGTAACTGAAGGATGTGGTGCTTATATTGCTGTTAAGCAAGAGATGATGCAGCCTACTGCTAGCATCAAAGACAGACCGGCACTTGCTATGATGGAAGATGCTGAGAAAAAGAATTTGATAACTCCTGGAAAGACAATTCTGATAGAGCCAACATCAGGCAACATGGGAATCAGTTTGGCATTTATGGCTGCCATGAAGGGATATAAGATGGTTTTGACTATGCCATCTTACACAAGCTTGGAAAGAAGGGTTTGTATGAGAGCATTTGGAGCTGAATTGATTCTCACTGATCCAACCAAAGGAATGGGAGGAACAGTGAAAAAGGCTTATGACCTTTTGGAATCCACACCAAATGCTTTTATGCTGCAACAATTTTCCAACCCAGCCAATACTAAGGTGCATTTCGAAACAACAGGTCCTGAGATATGGGAGGATACAAATGGACAAGTTGACATATTTGTGATGGGAATAGGTAGCGGAGGCACGGTTTCCGGGGTTGGACAATATCTTAAATCACAAAACCCTAATGTTAAGATATATGGAGTGGAGCCATCTGAAAGTAATGTGCTGAATGGTGGTAAACCTGGTCCTCATCAAATAACTGGAAATGGTGTTGGATTCAAACCAGACATACTGGACATGGATGTAATGGAAAAAGTCCTAGAAGTTAGCAGTGAAGATGCAGTTAACATGGCTAGGACATTGGCCTTGAAAGAAGGACTCATGGTTGGTATATCGTCTGGAGCAAACACAGTTGCAGCACTCAGATTGGCTAACCTACCTGAGAACAAAGGCAAACTGATTGTGGTAAACTTGAATTTCAGACTGTTCATCCAAGTTTCGGGGAACGATACCTGTCATCCGTCTTGTTCCAAGACCTTCGGACAGAAGCTGAAAACATGCAGCCAGTCTCGGTCGATTAATGTCTTCCATGAGTTCTCATACAACTAG |
|  |  | Protein | MASLMSFLKRTSSSSSSLAVACQHPMMMRRLMTTTATSVDSSSFAQRIRDLPKDLPGTNIKKHVSQLIGRTPLVYLNKVTEGCGAYIAVKQEMMQPTASIKDRPALAMMEDAEKKNLITPGKTILIEPTSGNMGISLAFMAAMKGYKMVLTMPSYTSLERRVCMRAFGAELILTDPTKGMGGTVKKAYDLLESTPNAFMLQQFSNPANTKVHFETTGPEIWEDTNGQVDIFVMGIGSGGTVSGVGQYLKSQNPNVKIYGVEPSESNVLNGGKPGPHQITGNGVGFKPDILDMDVMEKVLEVSSEDAVNMARTLALKEGLMVGISSGANTVAALRLANLPENKGKLIVVNLNFRLFIQVSGNDTCHPSCSKTFGQKLKTCSQSRSINVFHEFSYN* |
| *MsCSase36* | MS.gene41829.t1 | CDS | ATGGCAGAGAGAAATGCTTCTGCCATTAAGGTTGTTTCAAGAAAATCTTTATGGTTCTTTTTCACAACAACAACGTTATTTTTCATCATGTCATGGCTCTTTGTTCTCCGTTCCACACGTTCAGATTCAACACCAGCCACCATCTCACAGCTTCTTTCCACCTCCTCCAACGACGGTTCAAATTCCATAACATCTCAATCACAAAACTCTGAATCCTCCTTCAGCAATAGAGCAATCCTCGTAAACACCGAAACAAAAACACCAGAAACTCCAATATGCAAAACCGAAACAGCCACCATATCAAACGACGACACCAAGAGCAATAAAACTAAAGTTGTTTTGAAAATTTTCATGTATGATTTACCTCCCGAGTTTCATTTCGGGTTATTAGATTGGAAAGGCGATGAAAAGACGAAAAACGTTTGGCCTGATATGAAAACCAAGATACCACATTACCCTGGCGGTTTGAATTTGCAACACAGCATTGAATATTGGCTTACATTGGACATTCTTGCTTCAGAATTGCCAGAAATTTATCCTGCTAGAACTGTAATCAGAGTTAGAAACTCGACCGAAGCTGATGTTATATTTGTGCCATTTTTTTCATCTTTGACTTATAACAGACACTCCAAAACAGGTCCACGTGAAAAGAGGAGTAGAAACAAGGTGTTGCAAGAGAAGTTGGTGAGGTATTTGATGAGTCAAGAAGAATGGAAAAGGTCTGGTGGAAGGGATCATTTGATCTTGGCTCATCATCCTAATAGTATGTTGGATGCTAGAATGAAACTTTGGCCTGCAACTTTTATATTGTCTGATTTTGGGAGATATCCTCCTAATATAGCAAATGTTGATAAAGATGTGATTGCGCCGTATAAGCATGGAGGTTATGCTAGGCAAGAACTATTTTATCTTCTGAAAGAAGAAAAAGATGTACATTTTTCATTTGGGAGTGTACAGAAAGGTGGAGTGAGGAATGCAACAAATGGCATGCGATCTTCGAAATTCTGCCTCAACATAGCTGGCGACACGCCATCATCAAACCGCTTATTCGATGCCATTGCAAGTCACTGTGTTCCTGTCATAATCAGTGATGAAATTGAGCTTCCATATGAGGACGTTCTCGACTACTCCAAATTCTGTGTATTTGTTCGCACAAGGGATGCCGTTAAAATGAAGTATCTGATAAACTTCATTAGGAGTATTGGCAAAGATGAGTGGACAAGAATGTGGAACAGATTGAAAGAAGTTGAAAAATTCTTTGAATTTCAGTTCCCTTCTAAGGAAGGTGATGCTGTTCAAATGATTTGGCAAGCTGTTTCACAAACGAAGATAATGGCGCTATCATCGTCATCTTCTGCATCATGTTCATGCTTTTCACCTTCTACAACAATTCTTCCTCCTTCGCGTCCTTTCACTACTCAATTCCACCGTAACATCCGTTTCCGCCGAACAATTTCCGCAACCGCCACCGTCTCCACTCCGTCACTCTTCTCTACCACCAGAGAAGGACTTGACGCCGTTAACATTGCCGAAGATGTCACTCAGTTGATTGGAAATACACCGATGATATATCTGAATAAAGTTACAGATGGATGTGTAGCAAACGTTGCTGCAAAACTTGAATCCATGGAACCTTGCAGAAGTGTCAAGGATAGAATTGGCTATAGCATGTTAGCTGATGCTGAAGAGAGTGGAGCGATTTCTCCAGGGAAGACTGTTTTAGTTGAACCTACAACTGGAAATACGGGACTTGGCCTTGCTTTTGTTGCCGCAACAAAGGGTTATAAACTGATAGTCACAATGCCTGCTTCTGTTAATATTGAGAGGAGAATCCTTTTACGCACATTTGGTGCAGAGGTTGTTTTGACGAATGCGGAGAAGGGGCTGAAAGGGGCTGTTGACAAAGCTGAAGAAATTGTCTATGCCACACCCAATGCTTACATGTTTCGACAGTTTGATAACAGGAATAACACAAAGATCCACTTCGAAACTACAGGGCCTGAGATATGGGAGGATACAATGGGTAATGTTGATTTATTGGTTGCTGCTATTGGAACTGGCGGCACTATTACAGGCACTGGACAATATCTGAAAATGATGAACAAAAAAATAAAGGTGGTTGGGGTGGAACCTGCAGATAGAAGTATAATCTCAGGAGACAATCCAGGTTTCATACCAAGCATTTTGGATATCAAACTGCTTGATGAAGTCGTCAAGGTTACCAATGTTGAAGCTATTGAAATGGCAAGGAGATTAGCATTGGAAGAAGGATTACTGGTTGGGATTTCTTCAGGAGCTGCAGCAGCAGCTGCGATAACTTTAGCAAGACGGCCTGAAAATTCTGGAAAACTTATAGCGATGAAGAGTGGTTTATGTACTCCCCTTCACAGAAGTGGCAAATGA |
|  |  | Protein | MAERNASAIKVVSRKSLWFFFTTTTLFFIMSWLFVLRSTRSDSTPATISQLLSTSSNDGSNSITSQSQNSESSFSNRAILVNTETKTPETPICKTETATISNDDTKSNKTKVVLKIFMYDLPPEFHFGLLDWKGDEKTKNVWPDMKTKIPHYPGGLNLQHSIEYWLTLDILASELPEIYPARTVIRVRNSTEADVIFVPFFSSLTYNRHSKTGPREKRSRNKVLQEKLVRYLMSQEEWKRSGGRDHLILAHHPNSMLDARMKLWPATFILSDFGRYPPNIANVDKDVIAPYKHGGYARQELFYLLKEEKDVHFSFGSVQKGGVRNATNGMRSSKFCLNIAGDTPSSNRLFDAIASHCVPVIISDEIELPYEDVLDYSKFCVFVRTRDAVKMKYLINFIRSIGKDEWTRMWNRLKEVEKFFEFQFPSKEGDAVQMIWQAVSQTKIMALSSSSSASCSCFSPSTTILPPSRPFTTQFHRNIRFRRTISATATVSTPSLFSTTREGLDAVNIAEDVTQLIGNTPMIYLNKVTDGCVANVAAKLESMEPCRSVKDRIGYSMLADAEESGAISPGKTVLVEPTTGNTGLGLAFVAATKGYKLIVTMPASVNIERRILLRTFGAEVVLTNAEKGLKGAVDKAEEIVYATPNAYMFRQFDNRNNTKIHFETTGPEIWEDTMGNVDLLVAAIGTGGTITGTGQYLKMMNKKIKVVGVEPADRSIISGDNPGFIPSILDIKLLDEVVKVTNVEAIEMARRLALEEGLLVGISSGAAAAAAITLARRPENSGKLIAMKSGLCTPLHRSGK* |
| *MsCSase37* | MS.gene042525.t1 | CDS | ATGGCGCTATCATCGTCATCTTCTGCATCATGTTCATGCTTTTCACCTTCTACAACAATTCTTCCTCCTTCGCGTCCTTTCACTACTCAATTCCACCGTAACATCCGTTTCCGCCGAACAATTTCCGCAACCGCCACCGTCTCCACTCCGTCACTCTTCTCTACCACCAGAGAAGGACTTGACGCCGTTAACATTGCCGAAGATGTCACTCAGTTGCTTGGAAATACACCGATGATATATCTGAATAAAGTTACAGATGGATGTGTAGCAAACGTTGCTGCAAAACTTGAATCCATGGAACCTTGCAGAAGTGTCAAGGATAGAATTGGCTATAGCATGTTAGCTGATGCTGAAGAGAGTGGAGCAATTTCTCCGGGGAAGACTGTTTTAGTTGAACCTACAACTGGAAATACGGGACTTGGCCTTGCTTTTGTTGCCGCAACGAAGGGTTATAAACTGATAGTCACAATGCCTGCTTCTGTTAATATTGAGAGGAGAATCCTTTTACGAACATTTGGTGCAGAGGTTGTTTTGACGAATACGGAGAAGGGGCTGAAAGGGGCTGTTGACAAAGCTGAAGAAATTGTCTATGCCACACCCAATGCTTACATGTTTCGACAGTTTGATAACAGGAATAACACAAAGATCCACTTCGAAACTACAGGGCCAGAGATATGGGAGGATACAATGGGTAATGTTGATTTATTGGTTGCTGCTATTGGAACTGGCGGCACTATTACAGGCACGGGACAATATCTGAAAATGATGAACAAAAAAATAAAGGTGGTTGGGGTGGAACCTGCAGATAGAAGTATAATCTCAGGAGACAATCCAGGTTTCATACCAAGCATTTTGGATATCAAACTGCTTGATGAAGTTGTCAAGGTTACCAATGTTGAAGCTATTGAAATGGCAAGGAGATTAGCATTGGAAGAAGGATTACTGGTTGGGATTTCTTCAGGAGCTGCAGCAGCAGCTGCGATAACTTTAGCAAGACGGCCTGAAAATTCTGGAAAACTTATAGCGATGAAGAGTGGTTTCTGTGCTCCCCTTCACAAGTGGCAAATGATGATATGGTAA |
|  |  | Protein | MALSSSSSASCSCFSPSTTILPPSRPFTTQFHRNIRFRRTISATATVSTPSLFSTTREGLDAVNIAEDVTQLLGNTPMIYLNKVTDGCVANVAAKLESMEPCRSVKDRIGYSMLADAEESGAISPGKTVLVEPTTGNTGLGLAFVAATKGYKLIVTMPASVNIERRILLRTFGAEVVLTNTEKGLKGAVDKAEEIVYATPNAYMFRQFDNRNNTKIHFETTGPEIWEDTMGNVDLLVAAIGTGGTITGTGQYLKMMNKKIKVVGVEPADRSIISGDNPGFIPSILDIKLLDEVVKVTNVEAIEMARRLALEEGLLVGISSGAAAAAAITLARRPENSGKLIAMKSGFCAPLHKWQMMIW* |
| *MsCSase38* | MS.gene042523.t1 | CDS | ATGGCGCTATCATCGTCATCTTCTGCATCATGTTCATGCTTTTCACCTTCTACAACAATTCTTCCTCCTTCGCGTCCTTTCACTACTCAATTCCACCGTAACATCCGTTTCCGCCGAACAATTTCCGCAACCGCCACCGTCTCCACTCCGTCACTCTTCTCTACCACCAGAGAAGGACTTGACGCCGTTAACATTGCCGAAGATGTCACTCAGTTGCTTGGAAATACACCGATGATATATCTGAATAAAGTTACAGATGGATGTGTAGCAAACGTTGCTGCAAAACTTGAATCCATGGAACCTTGCAGAAGTGTCAAGGATAGAATTGGCTATAGCATGTTAGCTGATGCTGAAGAGAGTGGAGCAATTTCTCCGGGGAAGACTGTTTTAGTTGAACCTACAACTGGAAATACGGGACTTGGCCTTGCTTTTGTTGCCGCAACGAAGGGTTATAAACTGATAGTCACAATGCCTGCTTCTGTTAATATTGAGAGGAGAATCCTTTTACGAACATTTGGTGCAGAGGTTGTTTTGACGAATACGGAGAAGGGGCTGAAAGGGGCTGTTGACAAAGCTGAAGAAATTGTCTATGCCACACCCAATGCTTACATGTTTCGACAGTTTGATAACAGGAATAACACAAAGATCCACTTCGAAACTACAGGGCCAGAGATATGGGAGGATACAATGGGTAATGTTGATTTATTGGTTGCTGCTATTGGAACTGGCGGCACTATTACAGGCACGGGACAATATCTGAAAATGATGAACAAAAAAATAAAGGTGGTTGGGGTGGAACCTGCAGATAGAAGTATAATCTCAGGAGACAATCCAGGTTTCATACCAAGCATTTTGGATATCAAACTGCTTGATGAAGTTGTCAAGGTTACCAATGTTGAAGCTATTGAAATGGCAAGGAGATTAGCATTGGAAGAAGGATTACTGGTTGGGATTTCTTCAGGAGCTGCAGCAGCAGCTGCGATAACTTTAGCAAGACGGCCTGAAAATTCTGGAAAACTTATAGCG |
|  |  | Protein | MALSSSSSASCSCFSPSTTILPPSRPFTTQFHRNIRFRRTISATATVSTPSLFSTTREGLDAVNIAEDVTQLLGNTPMIYLNKVTDGCVANVAAKLESMEPCRSVKDRIGYSMLADAEESGAISPGKTVLVEPTTGNTGLGLAFVAATKGYKLIVTMPASVNIERRILLRTFGAEVVLTNTEKGLKGAVDKAEEIVYATPNAYMFRQFDNRNNTKIHFETTGPEIWEDTMGNVDLLVAAIGTGGTITGTGQYLKMMNKKIKVVGVEPADRSIISGDNPGFIPSILDIKLLDEVVKVTNVEAIEMARRLALEEGLLVGISSGAAAAAAITLARRPENSGKLIA* |
| *MsCSase39* | MS.gene83857.t1 | CDS | ATGGCGCTATCATCGTCATCTTCTGCATCATGTTCATGCTTTTCACCTTCTACAACAATTCTTCCTCCTTCGCGTCCTTTCACTACTCAATTCCACCGTAACATCCGTTTCCGCCGAACAATTTCCGCAACCGCCACCGTCTCCACTCCGTCACTCTTCTCTACCACCAGAGAAGGACTTGACGCCGTTAACATTGCCGAAGATGTCACTCAGTTGCTTGGAAATACACCGATGATATATCTGAATAAAGTTACAGATGGATGTGTAGCAAACGTTGCTGCAAAACTTGAATCCATGGAACCTTGCAGAAGTGTCAAGGATAGAATTGGCTATAGCATGTTAGCTGATGCTGAAGAGAGTGGAGCAATTTCTCCGGGGAAGACTGTTTTAGTTGAACCTACAACTGGAAATACGGGACTTGGCCTTGCTTTTGTTGCCGCAACGAAGGGTTATAAACTGATAGTCACAATGCCTGCTTCTGTTAATATTGAGAGGAGAATCCTTTTACGAACATTTGGTGCAGAGGTTGTTTTGACGAATACGGAGAAGGGGCTGAAAGGGGCTGTTGACAAAGCTGAAGAAATTGTCTATGCCACACCCAATGCTTACATGTTTCGACAGTTTGATAACAGGAATAACACAAAGATCCACTTCGAAACTACAGGGCCAGAGATATGGGAGGATACAATGGGTAATGTTGATTTATTGGTTGCTGCTATTGGAACTGGCGGCACTATTACAGGCACGGGACAATATCTGAAAATGATGAACAAAAAAATAAAGGTGGTTGGGGTGGAACCTGCAGATAGAAGTATAATCTCAGGAGACAATCCAGGTTTCATACCAAGCATTTTGGATATCAAACTGCTTGATGAAGTTGTCAAGGTTACCAATGTTGAAGCTATTGAAATGGCAAGGAGATTAGCATTGGAAGAAGGATTACTGGTTGGGATTTCTTCAGGAGCTGCAGCAGCAGCTGCGATAACTTTAGCAAGACGGCCTGAAAATTCTGGAAAACTTATAGCG |
|  |  | Protein | MALSSSSSASCSCFSPSTTILPPSRPFTTQFHRNIRFRRTISATATVSTPSLFSTTREGLDAVNIAEDVTQLLGNTPMIYLNKVTDGCVANVAAKLESMEPCRSVKDRIGYSMLADAEESGAISPGKTVLVEPTTGNTGLGLAFVAATKGYKLIVTMPASVNIERRILLRTFGAEVVLTNTEKGLKGAVDKAEEIVYATPNAYMFRQFDNRNNTKIHFETTGPEIWEDTMGNVDLLVAAIGTGGTITGTGQYLKMMNKKIKVVGVEPADRSIISGDNPGFIPSILDIKLLDEVVKVTNVEAIEMARRLALEEGLLVGISSGAAAAAAITLARRPENSGKLIA* |

**Table S4. List of syntenic gene pairs**

| **Gene Name** | **Gene ID** | **Gene Name** | **Gene ID** |
| --- | --- | --- | --- |
| *MsCSase04* | MS.gene005160.t1 | *MsCSase08* | MS.gene44030.t1 |
| *MsCSase04* | MS.gene005160.t1 | *MsCSase12* | MS.gene69913.t1 |
| *MsCSase04* | MS.gene005160.t1 | *MsCSase06* | MS.gene70799.t1 |
| *MsCSase05* | MS.gene80045.t1 | *MsCSase07* | MS.gene006455.t1 |
| *MsCSase05* | MS.gene80045.t1 | *MsCSase14* | MS.gene26887.t1 |
| *MsCSase05* | MS.gene80045.t1 | *MsCSase11* | MS.gene33576.t1 |
| *MsCSase06* | MS.gene70799.t1 | *MsCSase08* | MS.gene44030.t1 |
| *MsCSase06* | MS.gene70799.t1 | *MsCSase12* | MS.gene69913.t1 |
| *MsCSase07* | MS.gene006455.t1 | *MsCSase14* | MS.gene26887.t1 |
| *MsCSase07* | MS.gene006455.t1 | *MsCSase11* | MS.gene33576.t1 |
| *MsCSase07* | MS.gene006455.t1 | *MsCSase24* | MS.gene92293.t1 |
| *MsCSase08* | MS.gene44030.t1 | *MsCSase12* | MS.gene69913.t1 |
| *MsCSase09* | MS.gene44032.t1 | *MsCSase13* | MS.gene69911.t1 |
| *MsCSase11* | MS.gene33576.t1 | *MsCSase14* | MS.gene26887.t1 |
| *MsCSase15* | MS.gene26579.t1 | *MsCSase21* | MS.gene008717.t1 |
| *MsCSase15* | MS.gene26579.t1 | *MsCSase17* | MS.gene39577.t1 |
| *MsCSase15* | MS.gene26579.t1 | *MsCSase19* | MS.gene47527.t1 |
| *MsCSase16* | MS.gene26578.t1 | *MsCSase18* | MS.gene39578.t1 |
| *MsCSase16* | MS.gene26578.t1 | *MsCSase20* | MS.gene47526.t1 |
| *MsCSase17* | MS.gene39577.t1 | *MsCSase21* | MS.gene008717.t1 |
| *MsCSase17* | MS.gene39577.t1 | *MsCSase19* | MS.gene47527.t1 |
| *MsCSase18* | MS.gene39578.t1 | *MsCSase20* | MS.gene47526.t1 |
| *MsCSase19* | MS.gene47527.t1 | *MsCSase21* | MS.gene008717.t1 |
| *MsCSase24* | MS.gene92293.t1 | *MsCSase29* | MS.gene000153.t1 |
| *MsCSase24* | MS.gene92293.t1 | *MsCSase30* | MS.gene024871.t1 |
| *MsCSase24* | MS.gene92293.t1 | *MsCSase26* | MS.gene21844.t1 |
| *MsCSase25* | MS.gene37942.t1 | *MsCSase28* | MS.gene020786.t1 |
| *MsCSase25* | MS.gene37942.t1 | *MsCSase27* | MS.gene81336.t1 |
| *MsCSase26* | MS.gene21844.t1 | *MsCSase29* | MS.gene000153.t1 |
| *MsCSase26* | MS.gene21844.t1 | *MsCSase30* | MS.gene024871.t1 |
| *MsCSase27* | MS.gene81336.t1 | *MsCSase28* | MS.gene020786.t1 |
| *MsCSase31* | MS.gene013867.t1 | *MsCSase37* | MS.gene042525.t1 |
| *MsCSase31* | MS.gene013867.t1 | *MsCSase36* | MS.gene41829.t1 |
| *MsCSase32* | MS.gene023918.t1 | *MsCSase34* | MS.gene058651.t1 |
| *MsCSase36* | MS.gene41829.t1 | *MsCSase37* | MS.gene042525.t1 |
